# The EQ-5D-5L valuation study in Nigeria

**DOI:** 10.1007/s11136-026-04319-4

**Published:** 2026-06-15

**Authors:** Abdulrasheed Hassan Yusuf, Bello Usman Ardo, Montarat Thavorncharoensap, Bram Roudijk, Fredrick Dermawan Purba, Zhihao Yang, Meixia Liao, Usa Chaikledkaew, Sitaporn Youngkong, Ammarin Thakkinstian, Yakubu Adole Agada-Amade, Taiwo Gboluwaga Amole, Mohammed Nasir Sambo, Kelechi Ohiri

**Affiliations:** 1 Mahidol University Health Technology Assessment (MUHTA) Graduate Program, Mahidol University, Bangkok, Thailand; 2 National Health Insurance Authority (NHIA), Abuja, FCT Nigeria; 3 Social and Administrative Pharmacy Division, Department of Pharmacy, Faculty of Pharmacy, Mahidol University, Bangkok, Thailand; 4 EuroQol Research Foundation, Rotterdam, The Netherlands; 5 Department of Psychiatry, Erasmus University Medical Center, Rotterdam, The Netherlands; 6 Faculty of Psychology, Padjadjaran University, Bandung, Indonesia; 7 Health Services Management Department, Guizhou Medical University, Guiyang, China; 8 Saw Swee Hock School of Public Health, National University of Singapore, Singapore, Singapore; 9 Department of Clinical Epidemiology and Biostatistics, Faculty of Medicine Ramathibodi Hospital, Mahidol University, Bangkok, Thailand; 10 Department of Health Administration and Management, University of Nigeria, Enugu Campus, Enugu, Enugu State Nigeria; 11 Department of Community Medicine and Africa Center of Excellence for Population Health and Policy, Aminu Kano Teaching Hospital/Bayero University Kano, Kano, Kano State Nigeria; 12 Department of Community Medicine, Ahmadu Bello University Teaching Hospital, Zaria, Kaduna State Nigeria

**Keywords:** Health technology assessment, EQ-5D, Valuation study, Value set, Nigeria

## Abstract

**Purpose:**

A country-specific EQ-5D-5L value set ensures that health utility estimates reflect national preferences, enabling contextually appropriate health technology assessment (HTA) to inform efficient resource allocation decisions. This study aimed to develop the first EQ-5D-5L value set for Nigeria.

**Methods:**

Adult Nigerians were recruited from 12 states using multi-stage stratified quota sampling based on age, sex, and education. Face-to-face interviews were conducted through Computer-Assisted Personal Interviews using the EQ-PVT protocol. The interview comprises 2 main parts: composite time-trade-off (cTTO) and discrete choice experiment (DCE) tasks. The cTTO data were modelled using random intercept, Tobit, linear (heteroskedasticity-corrected), and Tobit (heteroskedasticity-corrected) models. DCE data were analyzed using Mixed Logit Model (MLM). Hybrid models combining the cTTO and DCE data were also estimated.

**Results:**

A total of 1,200 interviews were conducted. The Hybrid Tobit model with intercept, corrected for heteroscedasticity, and excluded flagged responses was considered the preferred model. The utility values of the best (11,111), 2nd best (21,111), worst (55,555), and 2nd worst (54,555) health states are 1, 0.963, − 0.733, and − 0.653, respectively. The most important dimension is Pain/Discomfort followed by Anxiety/Depression, Mobility, Usual Activity, and Self-care, respectively.

**Conclusion:**

This study provides the first EQ-5D-5L value set for Nigeria, derived from a representative adult population. This value set provides a strong foundation for HTA, supporting evidence-informed policy decisions and advancing progress towards Universal Health Coverage (UHC) in Nigeria and the wider West African region.

**Supplementary Information:**

The online version contains supplementary material available at https://doi.org/10.1007/s11136-026-04319-4.

## Abstract (plain language summary)

A country-specific EQ-5D-5L value set makes sure that health measurements reflect what matters most to people in that country. This study developed an EQ-5D-5L value set to measure how people in Nigeria value different health states. We interviewed 1200 adults from 12 states, ensuring a good balance of age, gender, and education levels. The most serious health problems perceived by Nigerians were related to pain/discomfort and anxiety/depression. This value set can facilitate the cost-effectiveness research, which could help guide health decisions in Nigeria and neighboring countries, supporting more efficient and fair use of resources as they work toward achieving Universal Health Coverage (UHC).

**Supplementary Information:**

The online version contains supplementary material available at https://doi.org/10.1007/s11136-026-04319-4.

## Introduction

Health Technology Assessment (HTA) is a multidisciplinary process that supports the achievement and sustainability of Universal Health Coverage (UHC) by informing evidence-based decision-making to promote an equitable, efficient, and high-quality health system [1, 2]. Economic evaluation, typically conducted as a cost-utility analysis (CUA), is an integral component of HTA, in which the costs of healthcare technologies are compared with their outcomes, usually measured as Quality-Adjusted Life Years (QALYs). QALY combines both quantity (i.e., length of life) and quality (i.e., quality of life or utility) of life into a single index measure, where one QALY means 1 year of life in perfect health [3].

The EQ-5D, developed by the EuroQol group in the 1990s [4], is the most widely recommended multi-attribute utility instrument (MAUI) by national HTA agencies for use in CUA [5]. These instruments comprise 5 dimensions: mobility (MO), self-care (SC), usual activities (UA), pain/discomfort (PD), and anxiety/depression (AD). For EQ-5D-5L, each dimension includes five levels of response (i.e., 1 = No Problems, 2 = Slight Problems, 3 = Moderate Problems, 4 = Severe Problems, and 5 = Extreme Problems/Unable to) [6]. The EQ-5D-5L value set refers to a set of utilities associated with each of the health states. Value sets are derived via valuation studies, which aim to measure people’s preferences for health. As the way people value health may differ across countries, value sets differ between countries [7]. A country-specific value set is highly recommended to ensure that decisions for resource allocation based on the economic evaluation reflect the health preferences of its population [7].

Nigeria is a country located in West Africa. With a population of 237.5 million, it is the most populous country in Africa and the sixth-most populous country in the world in 2025 [8]. While its Gross domestic product is the largest in Africa, its per capita income is low, and the distribution is inequitable [9]. Recently, the Nigerian government has put a substantial effort into achieving UHC and to reduce out-of-pocket spending through the Basic Health Care Provision Funds (BHCPF) scheme [10]. This aspiration of Nigeria emphasizes the crucial need for HTA evidence to ensure efficiency in the allocation and utilization of resources. However, it has been revealed that the lack of local data for conducting HTA research was among the major impediments to HTA implementation in the country [11].

At present, five EQ-5D-5L value sets (i.e., Uganda, Ethiopia, Egypt, Morocco, and Ghana) [12–17] and two EQ-5D-3L value sets (i.e., Zimbabwe and Tunisia) [18, 19] are available in Africa. Notably, Egypt, Morocco, and Tunisia are situated in the North African region. Ethiopia and Uganda are in East Africa. On the other hand, Zimbabwe is identified as part of the South African region, while Ghana is considered part of the West African region. Although Nigeria and Ghana are situated within the same region and share certain geo-demographic characteristics such as native language [20], they differ significantly in terms of cultural and demographic characteristics, healthcare system, and key health and economic indicators [21]. Nigeria is substantially larger than Ghana in terms of population, land area, and economic size. Additionally, the value set study in Ghana was derived from a small sample that exhibited a gender imbalance relative to the Ghanaian population [13]. Consequently, there is a compelling need to develop a country-specific value set for Nigeria. Hence, the objective of this study is to develop a Nigerian-specific EQ-5D-5L value set, contributing to the progress of HTA research in the country.

## Methods

### Setting

Nigeria is a diverse country with multiple ethnic groups and religious communities. Hausa, Yoruba, and Igbo represent the three most prominent ethnic groups in the country. The Hausa are predominantly Muslim (~ 95%), Igbo are almost entirely Christian (~ 98%), while Yoruba are more religiously mixed (~ 55% Muslim, ~ 35% Christian) [22, 23].

### Study protocol

Valuation was conducted using EuroQol Portable Valuation Technology (EQ-PVT v18), adapted from EQ-VT v2.1 [24, 25]. The protocol involves face-to-face interviews using computer-assisted software. The EQ-PVT is designed for offline administration so that subsequently the data can be extracted and analyzed. This is to overcome the challenges of poor internet connectivity. It has been used successfully in the Ethiopian EQ-5D-5L valuation study [16]. The protocol included two preference elicitation tasks: Composite Time-Trade-Off (cTTO) and Discrete Choice Experiment (DCE).

For the cTTO task, 86 health states were grouped into 10 blocks, and each respondent was randomly assigned one block consisting of 10 health states. Each block comprised 2 fixed health states (55,555, and one of the following five mild health states: 11,112, 11,121, 11,211, 12,111, and 21,111), together with 8 additional health states of varying level of severity [26]. The cTTO task is followed by a feedback module that displayed responses ranked from highest to lowest utility, allowing review and flagging of inconsistencies. The standard DCE task consists of 196 pairs of EQ-5D-5L health states grouped into 28 blocks [26], each block has 7 pairs of health states. However, in our study, a new DCE design consisting of 240 pairs of health states, grouped into 20 blocks was employed. Each respondent was randomly assigned to one block containing 12 pairs of health states [27].

Although English is the official language in Nigeria, there are 3 major languages spoken by the majority of the population (i.e., Hausa, Yoruba, and Igbo) [28]. English is also commonly used in the southern parts of Nigeria, especially among the Yoruba and Igbo communities. On the other hand, Hausa predominates as the primary language of communication in the northern region, reflecting the linguistic preferences of the Hausa ethnic group. Therefore, English and Hausa versions of the EQ-PVT were used for this study, given that English and Hausa versions of the EQ-5D-5L are currently available for Nigeria.

### Study population and sampling technique

Adults (≥ 18 years) able to complete valuation tasks were eligible; acutely ill individuals, those unable to understand tasks, or unwilling to consent were excluded. Following international guidance, the minimum required sample was 1000 [26, 29]. We increased the sample size by 20% to cover possible incomplete, missing, or invalid responses. Therefore, the total sample size for this study was 1200 respondents.

To obtain a nationally representative sample of the Nigerian population, a stratified multi-stage quota sampling method was used. The sampling method was modified from the Nigeria Living Standards Survey (NLSS) 2018–2019 conducted by the National Bureau of Statistics (NBS) [30]. Firstly, the 36 states and the FCT were stratified into 6 geopolitical zones (i.e., North-West, North-East, North-Central, South-West, South-East, and South-South). It is noteworthy that states within the same zone tend to share similar characteristics, particularly in terms of ethnic composition, religion, and cultural norms [31]. Then, 2 states were randomly selected from each zone. For each selected state, 10 Enumeration Areas (EAs) were later selected from the EAs national master sample based on systematic random sampling. The EAs master sample, consisting of 200 pre-sampled EAs for each state, was developed by the NBS. This master sample is recommended as a sampling frame for the national surveys [30]. EAs affected by insurgencies such as Boko Haram, banditry/kidnappings, and Biafran agitations [32, 33] or EAs with very few residential households were excluded from the study. The sampling strategy is illustrated in Supplementary Fig. 1.

The total number of respondents sampled in each state was proportionate to the size of the adult population in that state. For each EA, respondents were selected based on quota sampling to obtain a representation of age group (i.e., 15–24, 25–34, 35–44, 45–54, 55–64, 65–74, and 75 + year), gender (i.e., male and female), and educational status (i.e., no formal education, primary education, secondary education, tertiary education, and others) that reflect the national demographics. After arriving at the selected EA, a brief household listing was carried out to identify all households within the EA, which served as the sampling frame. From this list, 10 households were selected using systematic random sampling. Within each selected household, one respondent was chosen according to predefined quota criteria. If more than one individual met the criteria, the oldest person was selected. If the required sample size was not achieved from the initially selected 10 households, additional households were selected sequentially from the list. This process continued until the target sample size for each EA was reached.

### Data collection

Data was collected face-to-face by Computer-Assisted Personal Interviews (CAPI) using the EQ-PVT software between August and December 2024. The interview was conducted by 12 health professionals from the NHIA with oversight provided by 5 supervisors. Training included a five-day workshop followed by a pilot interview, during which each interviewer conducted 15 interviews. The training workshop also included presentations on basic information about the EQ-5D-5L and its value set, preference elicitation methods, the quality control (QC) process, handling the software, and an EQ-PVT practice session.

Ethical approval was obtained from the National Health Research Ethics Committee of Nigeria (NHREC/01/01/2007) and Mahidol University IRB (No.78.0319/EC.0447). Written informed consent was obtained, and respondents received 3,000 Naira (~ USD 2) compensation.

### Quality control (QC)

Standard EuroQol QC procedures were applied [34, 35]. Interviews were tagged suspicious and flagged if: (1) the “worse than dead (WTD)” task was not explained in the wheelchair examples, (2) < 3 min spent on the wheelchair example, (3) clear inconsistencies in cTTO responses, or (4) all 10 cTTO tasks completed in < 5 min. Data collection was conducted in three phases, with interim QC and analysis carried out between each phase.

### Modeling and data analysis

Twenty-parameters were generated [17, 36]. These parameters represent the disutility for each dimension starting from Mobility level 2 (MO2) to Anxiety & Depression level 5 (AD5).

For cTTO, random intercept, Tobit, linear (heteroskedasticity-corrected), and Tobit (heteroskedasticity-corrected) models were tested. Tobit was appropriate given censoring at − 1 and 1, while heteroskedasticity models addressed variation in cTTO values by health state severity. Each model was run with/without intercepts and flagged responses.

The DCE data were analyzed using the Conditional Logistic Model (CLM) and the Mixed Logistic Model (MLM) [37]. Compared to the CLM, the MLM has the additional advantage of accounting for preference heterogeneity, thereby providing a more valid description of the data. Finally, a hybrid model combining the continuous data from the cTTO and dichotomous data from the DCE was explored.

The model selection was based on logical consistency (i.e., positive monotonous disutility across the coefficients of the model with higher levels within each dimension corresponded to larger coefficients), measures of goodness of fit including Akaike Information Criterion (AIC) and Bayesian Information Criterion (BIC), predictive accuracy measured by Mean Absolute Errors (MAE) for the means of the 86 states included in the cTTO design, and parsimony. Dimension importance was ranked by the value of level 5 coefficient. Analyses were conducted in Stata SE, version 18.0.

## Results

### Sample characteristics

Of the 1200 respondents interviewed, 3 were missing due to technical problems, resulting in a final dataset of 1,197 respondents for analysis. The median age of the sample was 34 years, with an Inter-Quartile Range (IQR) of 22 years. The sample has similar characteristics to the Nigerian adult population in terms of age, gender, education, ethnicity, and religion, as shown in Table 1. Only 174 (14.6%) of the respondents completed the interview using the Hausa version of the EQ-PVT, while the others opted for the English version.

**Table 1:** Sample characteristics

Study sample (n = 1197)			Nigerian adults’ population	
Gender	N	%	Gender [30]	%
Male	594	49.6	Male	49.2
Female	603	50.4	Female	50.8
Age group (years)			Age group (years) [30]	
18–24	370	30.9	18–24	30.7
25–34	256	21.4	25–34	21.7
35–44	220	18.4	35–44	17.9
45–54	148	12.4	45–54	12.7
55–56	95	7.9	55–56	8.2
65–74	69	5.8	65–74	5.4
75 +	39	3.3	75 +	3.5
Education			Education [30]	
No School	213	17.8	No School	19.1
Primary	252	21.1	Primary	20.8
Secondary	388	32.4	Secondary	31.6
Tertiary	150	12.5	Tertiary	12.0
Religious/Others	194	16.2	Others	16.6
Ethnicity			Ethnicity [28]	
Hausa	341	28.5	Hausa	30.0
Igbo	179	15.0	Igbo	15.2
Yoruba	178	14.9	Yoruba	15.5
Others	499	41.7	Others	39.3
Religion			Religion [28]	
Christianity	576	48.1	Christianity	45.9
Islam	616	51.5	Islam	53.5
Traditional	5	0.4	Traditional	0.6
*Occupation*				
Housewife	104	8.7		
Student	147	12.3		
Retired	25	2.1		
Not working	69	5.8		
Employed	117	9.8		
Self employed	735	61.4		
*Marital status*				
Single	513	42.9		
Married	632	52.8		
Divorced	14	1.2		
Widowed	35	2.9		
Others	3	0.3		

### QC report

**Fig. 1:**
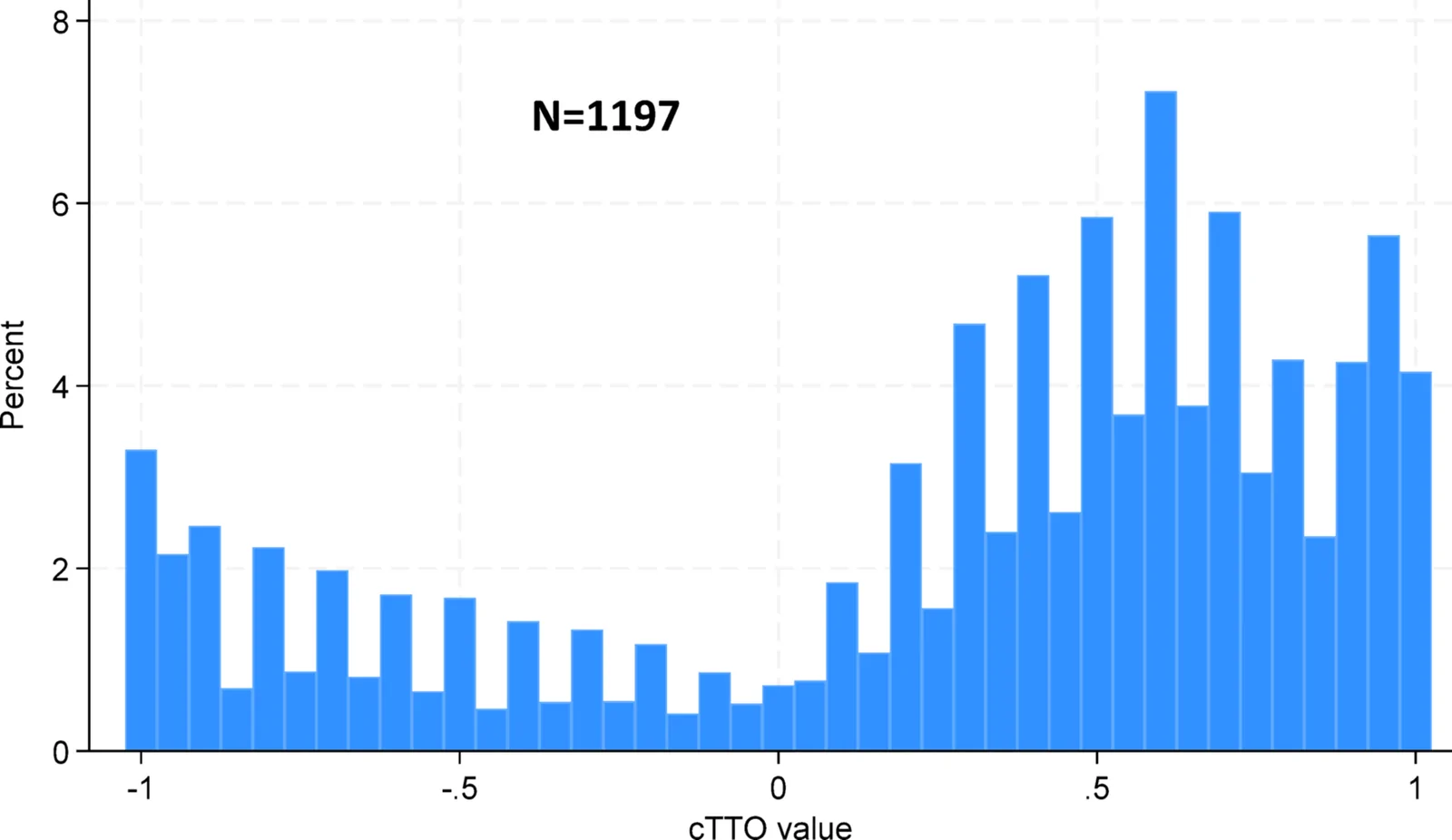
Distribution of cTTO observations by value

**Fig. 2:**
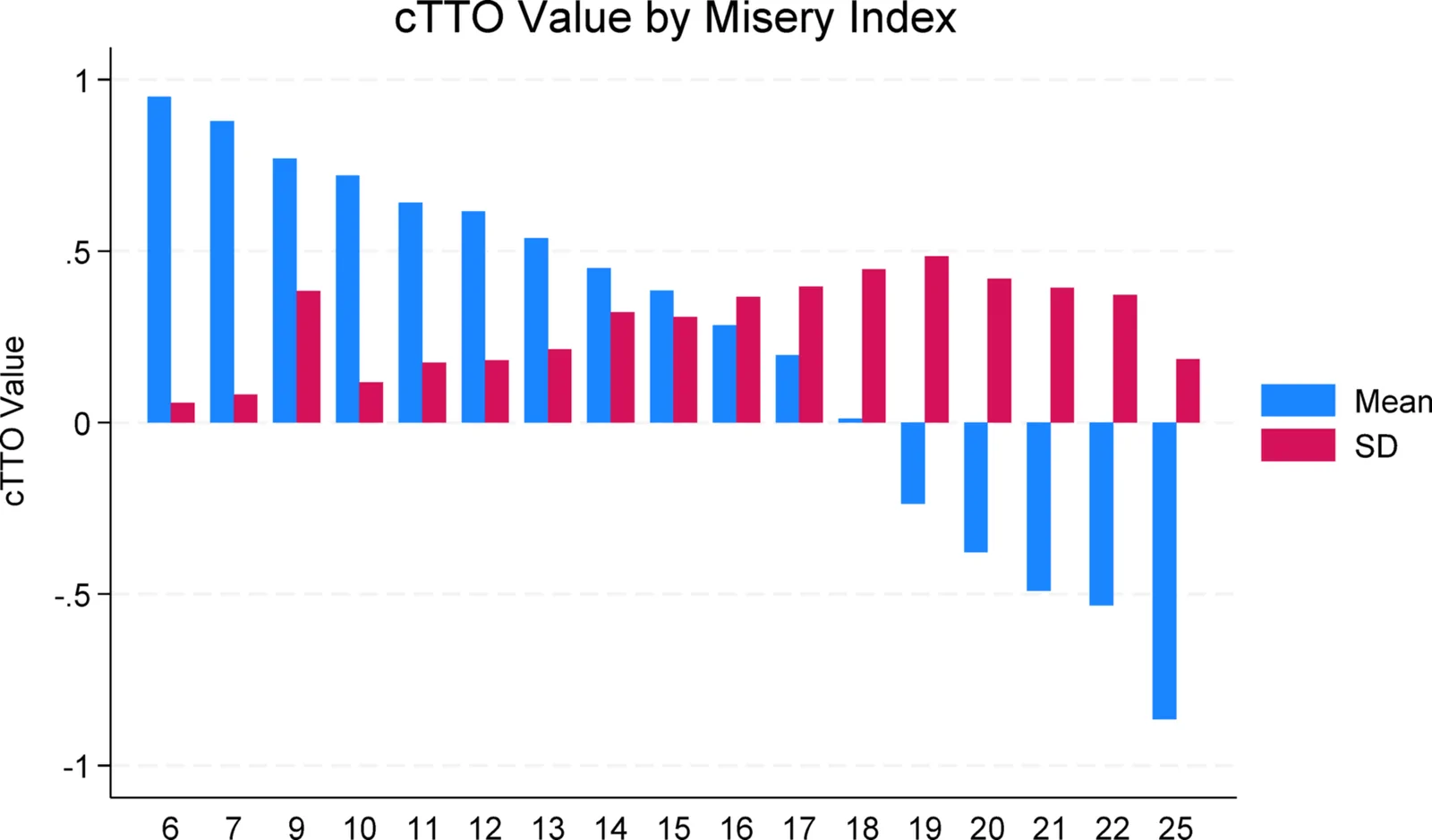
Mean and SD of cTTO Value by level sum score (Misery Index)

Mean time spent on the cTTO task was 31.57 ± 8.68 min. The distribution of cTTO observations shows slight clustering of values around 0.5 and 1, as shown in Fig. 1. The variance of the cTTO values increased with the severity of the health states, see Fig. 2. Eight interviews were flagged from the QC report. These include two flags for spending < 3 min on wheelchair examples, three flags for spending < 5 min on the 10 cTTO task, one flag for not explaining the WTD task, and two flags for severe inconsistencies involving 55,555 health state. From the feedback module, 392 (3.3%) responses were flagged by the respondents. Altogether, combined flagged responses represented approximately 4% of the total cTTO data.

### Modelling result

A total of 34 distinct models were estimated, comprising 16 cTTO models, 2 DCE models, and 16 hybrid models. Models 1–4 represented cTTO models incorporating all responses and an intercept term, while Models 5–8 did not include flagged responses but retained the intercept. Models 9–12 included all cTTO responses but omitted the intercept, while models 13–16 excluded flagged responses and did not include an intercept. DCE models were model 17–18 (i.e., CLM, MLM). Models 19–22 were hybrid models (i.e., cTTO and CLM models) with all responses and intercept, while models 23–26 are hybrid models (i.e., cTTO and CLM models) without flagged responses but retained the intercept. Lastly, models 27–30 were hybrid models (i.e., cTTO and CLM) with all responses but did not include intercept, and models 30–34 were hybrid models without flagged responses and omitted the intercept.

Given that utility values ranged from -1 to 1, and evidence of heteroskedasticity was observed, cTTO model based on Tobit regression, censored at -1 and adjusted for heteroskedasticity (i.e., models 4, 8, 12, and 16), along with four hybrid models (i.e., models 22, 26, 30, and 34) were selected as the candidate models (Table 2). Six of the candidate models—models 4, 8, 12, 16, 22, and 26—demonstrated logical consistency, exhibiting significantly greater value decrements for more severe health problems (Table 2). Among the four candidate cTTO models (i.e., models 4, 8, 12, and 16), the model that excluded flagged responses while including an intercept (model 4) demonstrated greater predictive accuracy. This was evidenced by lower MAE in predicting the 86 health states included in the cTTO task design. Similarly, among the hybrid models, model 26—which excluded flagged responses but retained an intercept—exhibited higher predictive accuracy (i.e., lower MAEs) compared to model 22. Details of the 34 models are reported in Supplementary Table 1.

**Table 2:** Characteristics of candidate models

Independent Variable	Tobit/heteroskedastic cTTO models without Flagged responses						Tobit/heteroskedastic cTTO models with all responses					
	With intercept (Model 8)			Without intercept (Model 16)			With intercept (Model 4)			Without intercept (Model 12)		
	Coefficient	SE	P- Value	Coefficient	SE	P- Value	Coefficient	SE	P- Value	Coefficient	SE	P- Value
*Mobility (MO)*												
MO2	0.021	0.005	< 0.001	0.033	0.004	< 0.001	0.018	0.005	0.001	0.031	0.005	< 0.001
MO3	0.054	0.009	< 0.001	0.059	0.009	< 0.001	0.051	0.009	< 0.001	0.056	0.009	< 0.001
MO4	0.226	0.010	< 0.001	0.230	0.010	< 0.001	0.226	0.010	< 0.001	0.231	0.010	< 0.001
MO5	0.303	0.009	< 0.001	0.302	0.009	< 0.001	0.296	0.008	< 0.001	0.296	0.008	< 0.001
*Self-Care (SC)*												
SC2	0.030	0.005	< 0.001	0.043	0.004	< 0.001	0.032	0.005	< 0.001	0.045	0.004	< 0.001
SC3	0.067	0.007	< 0.001	0.066	0.007	< 0.001	0.069	0.007	< 0.001	0.068	0.007	< 0.001
SC4	0.248	0.009	< 0.001	0.251	0.009	< 0.001	0.245	0.009	< 0.001	0.248	0.009	< 0.001
SC5	0.255	0.008	< 0.001	0.254	0.008	< 0.001	0.257	0.008	< 0.001	0.256	0.008	< 0.001
*Usual Activity (UA)*												
UA2	0.034	0.005	< 0.001	0.047	0.004	< 0.001	0.034	0.005	< 0.001	0.047	0.004	< 0.001
UA3	0.079	0.008	< 0.001	0.086	0.007	< 0.001	0.080	0.007	< 0.001	0.088	0.007	< 0.001
UA4	0.198	0.008	< 0.001	0.206	0.008	< 0.001	0.199	0.008	< 0.001	0.207	0.008	< 0.001
UA5	0.250	0.008	< 0.001	0.253	0.008	< 0.001	0.249	0.008	< 0.001	0.252	0.008	< 0.001
*Pain/Discomfort (PD)*												
PD2	0.040	0.004	< 0.001	0.050	0.003	< 0.001	0.038	0.004	< 0.001	0.049	0.004	< 0.001
PD3	0.059	0.008	< 0.001	0.058	0.008	< 0.001	0.057	0.008	< 0.001	0.056	0.008	< 0.001
PD4	0.274	0.009	< 0.001	0.269	0.009	< 0.001	0.269	0.008	< 0.001	0.264	0.008	< 0.001
PD5	0.470	0.010	< 0.001	0.480	0.009	< 0.001	0.465	0.009	< 0.001	0.475	0.009	< 0.001
*Anxiety/Depression (AD)*												
AD2	0.042	0.004	< 0.001	0.052	0.003	< 0.001	0.040	0.004	< 0.001	0.050	0.003	< 0.001
AD3	0.076	0.008	< 0.001	0.084	0.008	< 0.001	0.075	0.008	< 0.001	0.084	0.008	< 0.001
AD4	0.218	0.008	< 0.001	0.221	0.008	< 0.001	0.216	0.008	< 0.001	0.220	0.008	< 0.001
AD5	0.361	0.007	< 0.001	0.364	0.007	< 0.001	0.360	0.007	< 0.001	0.363	0.007	< 0.001
Intercept/Constant	0.019	0.005	< 0.001	-	-	-	0.020	0.005	< 0.001	-	-	-
AIC	516			531			860			878		
BIC	824			833			1171			1181		
MAE (All states)	0.221			0.221			0.223			0.224		
MAE (86 states)	0.0619			0.0632			0.0612			0.0625		
Order of importance	PD > AD > MO > SC > UA			PD > AD > MO > SC > UA			PD>AD>MO>SC>UA			PD>AD>MO>SC>UA		

Independent Variable	Tobit/heteroskedastic Hybrid models without Flagged responses						Tobit/heteroskedastic Hybrid models with all responses					
	With intercept (Model 26)			Without intercept (Model 34)			With intercept (Model 22)			Without intercept (Model 30)		
	Coefficient	SE	P- Value	Coefficient	SE	P- Value	Coefficient	SE	P- Value	Coefficient	SE	P- Value
*Mobility(MO)*												
MO2	**0.028**	**0.004**	** < 0.001**	**-0.003**	0.006	**0.660**	0.026	0.004	< 0.001	**-0.006**	0.006	**0.324**
MO3	**0.090**	**0.005**	** < 0.001**	0.080	0.006	< 0.001	0.087	0.005	< 0.001	0.078	0.006	< 0.001
MO4	**0.197**	**0.005**	** < 0.001**	0.206	0.007	< 0.001	0.195	0.005	< 0.001	0.202	0.007	< 0.001
MO5	**0.335**	**0.005**	** < 0.001**	0.368	0.007	< 0.001	0.332	0.005	< 0.001	0.364	0.007	< 0.001
*Self-Care (SC)*												
SC2	**0.031**	**0.003**	** < 0.001**	0.005	0.006	**0.410**	0.032	0.003	< 0.001	0.006	0.006	**0.276**
SC3	**0.084**	**0.005**	** < 0.001**	0.086	0.006	< 0.001	0.084	0.005	< 0.001	0.087	0.006	< 0.001
SC4	**0.183**	**0.005**	** < 0.001**	0.201	0.007	< 0.001	0.182	0.005	< 0.001	0.200	0.007	< 0.001
SC5	**0.263**	**0.005**	** < 0.001**	0.297	0.007	< 0.001	0.265	0.005	< 0.001	0.300	0.007	< 0.001
*Usual Activity (UA)*												
UA2	**0.031**	**0.003**	** < 0.001**	**-0.003**	0.006	**0.655**	0.031	0.003	< 0.001	**-0.002**	0.006	**0.694**
UA3	**0.078**	**0.004**	** < 0.001**	0.073	0.006	< 0.001	0.079	0.004	< 0.001	0.074	0.006	< 0.001
UA4	**0.188**	**0.005**	** < 0.001**	0.198	0.007	< 0.001	0.189	0.005	< 0.001	0.198	0.007	< 0.001
UA5	**0.282**	**0.005**	** < 0.001**	0.302	0.006	< 0.001	0.283	0.005	< 0.001	0.301	0.006	< 0.001
*Pain/Discomfort (PD)*												
PD2	**0.045**	**0.003**	** < 0.001**	0.039	0.006	0.039	0.044	0.003	< 0.001	0.038	0.006	< 0.001
PD3	**0.115**	**0.004**	** < 0.001**	0.123	0.006	0.123	0.112	0.005	< 0.001	0.122	0.006	< 0.001
PD4	**0.288**	**0.005**	** < 0.001**	0.320	0.007	0.320	0.285	0.005	< 0.001	0.318	0.006	< 0.001
PD5	**0.479**	**0.006**	** < 0.001**	0.538	0.007	0.538	0.477	0.006	< 0.001	0.539	0.007	< 0.001
*Anxiety/Depression (AD)*												
AD2	**0.041**	**0.003**	** < 0.001**	0.013	0.006	0.023	0.039	0.003	< 0.001	0.011	0.006	**0.052**
AD3	**0.087**	**0.005**	** < 0.001**	0.079	0.007	< 0.001	0.086	0.005	< 0.001	0.076	0.007	< 0.001
AD4	**0.207**	**0.005**	** < 0.001**	0.227	0.006	< 0.001	0.206	0.005	< 0.001	0.225	0.006	< 0.001
AD5	**0.365**	**0.005**	** < 0.001**	0.393	0.007	< 0.001	0.365	0.005	< 0.001	0.392	0.007	< 0.001
Intercept/Constant	**0.009**	**0.002**	** < 0.001**	-	-	-	0.010	0.002	< 0.001	-	-	-
AIC	**19,623**			25,555			20,089			26,027		
BIC	**19,974**			25,890			20,441			26,362		
MAE (All states)	0.220						0.221					
MAE (86 states)	**0.0647**			0.0671			0.0737			0.0682		
Order of importance	**PD > AD > MO > UA > SC**			PD > AD > MO > UA > SC			PD>AD>MO>UA>SC			PD>AD>MO>UA>SC		

AIC: Akaike Information Criterion, BIC: Bayesian Information Criterion, MAE: Mean Absolute Error, MO: mobility, SC: self−care, UA: usual activities, PD: pain/discomfort, AD: Anxiety/depression, TTO: time−trade off, SE: Standard error,

For DCE data, both CLM and MLM (i.e., models 17–18) were logically consistent. A Bland–Altman plot and scatterplots constructed to assess the level of agreement between the cTTO and the DCE models showed that the two data sets have good agreement between them (See Supplementary Fig. 2–3). In all 34 models, PD was the most important dimension. However, there are some disparities in the order of importance of the other four dimensions. For all the four candidate cTTO models (i.e., models 4, 8, 12, and 16), the order of importance is PD > AD > MO > SC > UA while for the DCE data, the order is PD > MO > AD > UA > SC for CLM (model 17) and PD > AD > MO > UA > SC for MLM (model 18). In the two candidate hybrid models (22 and 26), the order is similar with the MLM (PD > AD > MO > UA > SC). A summary of model performance and order of importance of all candidate models are provided in Supplementary Table 2.

While models 22 and 26 are highly similar, as both are logically consistent Tobit models with an intercept and corrected for heteroskedasticity, the final model chosen is model 26. In model 26, flagged responses were excluded, as they are expected to be of relatively poor quality. Furthermore, MAE across all 3,125 states (0.220 vs. 0.221), and MAE for the 86 health states included in the cTTO design (0.0647 vs. 0.0737) were all slightly lower for model 26 compared with model 22, indicating better predictive accuracy of model 26. For these reasons, model 26 was selected as the preferred model. While comparatively lower AIC and BIC values were also observed for model 26, these were not the primary basis for selecting model 26 over model 22. As AIC and BIC depend on sample size—typically decreasing as sample size decreases—the smaller values observed for model 26 do not necessarily imply a better fit, particularly in light of the fact that the two models were estimated using different sample sizes.

In the preferred model, all the coefficients are monotonous and logically consistent (i.e., they are all positive and increase with increasing severity from level 2 to level 5 in all five dimensions). The intercepts represent baseline disutility for all the predicted 3125 health states, excluding 11,111, which has a utility value of 1. The health state with the second highest predicted utility is 21,111 (0.963), whereas 55,555 has the lowest predicted utility of -0.733, followed by 54,555 with the second lowest utility value of -0.653.

The mathematical representation of the preferred model 26 for health state X is given by the expression: utility (health state X) = 1- (0.009*N1 + 0.028* mo2 + 0.090*mo3 + 0.197* mo4 + 0.335*mo5 + 0.031*sc2 + 0.084*sc3 + 0.183*sc4 + 0.263*sc5 + 0.031*ua2 + 0.078*ua3 + 0.188*ua4 + 0.282*ua5 + 0.045*pd2 + 0.115*pd3 + 0.288*pd4 + 0.479*pd5 + 0.041*ad2 + 0.087*ad3 + 0.207*ad4 + 0.365*ad5).

where N1 is a dummy variable which shows the additional disutility of not being in state 11,111. Therefore, 0.009 must be subtracted while calculating the utilities of all health states except 11,111. Example of calculating utility for 23,455 is shown below.

Utility of 23,455 = 1−Intercept−mo2−sc3−ua4−pd5—ad5.

=1−0.009−0.028−0.084−0.188−0.479−0.365 = −0.153

In addition, we compared the cTTO models for the Hausa (i.e., Tobit corrected for heteroskedasticity with intercept and including flagged responses: Model 35) and English versions to identify any significant differences in the model coefficients (Supplementary Table 3). Results showed that the coefficients for the Hausa version were all logically consistent. No significant difference in terms of coefficient values from the 2 versions was observed except for MO4, MO5, PD2, and PD4, in which the coefficient values for Hausa were significantly lower.

The order of importance of the dimensions obtained from the Hausa version was PD > AD > MO > SC > UA, which was similar to the cTTO models for the overall sample (Model 4: Tobit corrected for heteroskedasticity with intercept and including flagged responses). Nevertheless, it is important to note that the small sample size for the Hausa version restricted a comprehensive examination of the hybrid model. The mean TTO Values of the 86 health states were presented in Supplementary Table 4.

## Discussion

This study developed the first EQ-5D-5L value set for Nigeria using a nationally representative adult sample and the standard EQ-PVT protocol. Quality control showed that fewer than 4% of cTTO responses were flagged, which is lower than the proportion observed in many other studies [38]. While EuroQol QC does not formally cover DCE data, both CLM and MLM analyses produced logically consistent results.

About 3.3% of cTTO responses were censored at –1 reflecting participants willing to trade all available years to avoid the most severe health states. This pattern is common in other countries, including the Netherlands [39], Spain [40], Germany [41], Portugal [42], Poland [43], Hungary [44], France [45], Denmark [46], USA [47], Peru [48], Thailand [36], Indonesia [49], Hong Kong [50], Taiwan [51], Vietnam [52], Ethiopia [16], and Uganda [15], was accounted for in our Tobit-based modeling. Consistent with international findings [15–17, 36, 40–42, 44–47, 49, 53, 54], our cTTO data exhibited heteroskedasticity, with variance increasing in more severe states, justifying the use of heteroskedastic models.

Our final value set was estimated using a hybrid Tobit model corrected for heteroskedasticity with an intercept and excluded flagged responses (model 26). Hybrid models are widely adopted internationally because they maximize data utilization and combine the complementary strengths of cTTO and DCE [16, 36, 40–46, 49–53, 55, 56]. In some countries, such as the Netherlands [39], Canada [57], Hungary [44], Mexico [54], USA [47], Peru [48], Egypt [17], and Uganda [15], however, only cTTO data were used to develop the EQ-5D-5L value set.

The order of importance across the dimensions was PD > AD > MO > UA > SC. PD emerged as the most important dimension, consistent with studies from Canada [57], England [55], Germany [41], Portugal [42], Poland [43], France [45], USA [47], Uruguay [58], Mexico [47] Vietnam [52], and Uganda [15]. While SC was the least important in Nigeria, UA was the least important in the Netherlands, the USA, and several Asian settings [39, 47, 51, 52, 59, 60]. Based on these findings, Nigerian health authorities may consider prioritizing interventions that address pain/discomfort and mental health, as these dimensions were identified as most important by the Nigerian population.

In our study, the values of coefficients rose sharply between levels 3 and 4 across all the dimensions, indicating that Nigerians attach substantially higher disutility to severe and extreme problems than to mild or moderate ones. Also, the bigger standard errors of the higher-level coefficients indicate higher uncertainty around severe states.

Compared with other African countries, Nigeria’s utility range (− 0.733 to 1) was similar to that of Ethiopia (− 0.718 to 0.974), which also employed the EQ-PVT protocol and hybrid modelling [16]. In contrast, the Ugandan valuation study, which applies the EQ-VT Lite protocol and hybrid modelling, had a wider range (− 1.116 to 1) [15], while Ghana’s value set, which was also generated using the EQ-VT Lite protocol and hybrid modelling, had a narrower range (− 0.493 to 1) [13]. Dimension ranking also varied: SC was the least important in both Nigeria and Ethiopia, whereas AD ranked as the least important in Uganda, and UA was deemed the least important in Ghana. Like our study, Uganda also found PD as the most important dimension, but Ethiopia and Ghana found varying results, where AD and MO were the most important dimensions, respectively. These differences highlight cross-country variability in health preferences and reinforce the importance of developing country-specific value sets.

When comparing the cTTO models between the English and the Hausa versions, the relative ordering of the dimension coefficients was similar. However, the coefficients for MO4, MO5, PD2, and PD4 were significantly lower in the Hausa version than in the English version, whereas the constant term was only slightly higher in the Hausa model. This suggests that, for more severe health states in the PD and MO dimensions, the Hausa version may yield higher utility values. Nevertheless, during data collection, we observed that even in predominantly Hausa-speaking regions, most respondents preferred to be interviewed in English. Additionally, the comparison of the English and Hausa samples showed a significant difference in several socio-demographic characteristics (See Supplementary Table 5). Furthermore, it should be acknowledged that the study was not sufficiently powered to formally assess differences between the value sets of the two language versions. In addition, given that the analysis involved testing several hypotheses for all 20 parameters (i.e., Δmo2 to Δad5), and acknowledging the limitation that a Bonferroni correction could not be applied, any statistically significant findings should be interpreted with caution. Thus, at present, the value set derived from the overall population, irrespective of language or ethnicity, is preferred until more robust evidence becomes available to clarify potential differences between value sets obtained from the two language versions.

Our study has notable strengths. It was conducted in Africa’s most populous country with a demographically representative sample, enhancing generalizability and relevance for health policy. The findings also provide a resource for neighboring countries lacking their own value sets. However, limitations include the use of only English and Hausa versions of EQ-PVT, which may have excluded some local language groups, though these are the two most widely spoken languages nationally [61]. In addition, we did not estimate a cTTO–MLM hybrid model due to methodological complexity. It should also be noted that cTTO-MLM models are not possible to estimate in standard econometric software packages such as Stata, and require tailor-made implementations in e.g., Bayesian software packages. To date, no previous value set studies have applied a cTTO-MLM approach in their analyses. Although we did not employ such a model, our chosen model demonstrated good performance. Nevertheless, future work could be done to explore this approach.

## Conclusions

This study established the first Nigerian EQ-5D-5L value set using a hybrid model that combined cTTO and DCE data. The value set provides a robust foundation for health economic evaluations and will strengthen evidence-based resource allocation in Nigeria. It also offers a reference for West African countries seeking to advance UHC through locally relevant HTA.

## Supplementary Information

Below is the link to the electronic supplementary material.

Supplementary Material 1.

## Data Availability

The data supporting the findings of this study are available from the corresponding author upon reasonable request.
