# Supplementary material for: The EQ-5D-5L valuation study in Nigeria
Source: Qual Life Res. 2026 Jun 15;35(8):206. doi: 10.1007/s11136-026-04319-4 (PMC13269515; doi:10.1007/s11136-026-04319-4)
Supplement: Supplementary file 1 — Supplementary Material 1. [file 11136_2026_4319_MOESM1_ESM.docx]

**The EQ-5D-5L Valuation Study in Nigeria**

Abdulrasheed Hassan Yusuf^1,2^, Bello Usman Ardo^1,2^, Montarat Thavorncharoensap^1,3*^, Bram Roudijk^4,5^, Fredrick Dermawan Purba^6^, Zhihao Yang^7^, Meixia Liao^8^, Usa Chaikledkaew^1,3^, Sitaporn Youngkong^1,3^, Ammarin Thakkinstian^1,9^, Yakubu Agada-Amade^2,10^, Taiwo Globuwaga Amole^11^, Mohammed Nasir Sambo^12^, Kelechi Ohiri^2^

^1^ Mahidol University Health Technology Assessment (MUHTA) Graduate Program, Mahidol University, Bangkok, Thailand

^2^ National Health Insurance Authority (NHIA), Abuja FCT, Nigeria

^3^ Social and Administrative Pharmacy Division, Department of Pharmacy, Faculty of Pharmacy, Mahidol University, Bangkok, Thailand

^4^ EuroQol Research Foundation, Rotterdam, the Netherlands

^5^ Erasmus University Medical Center, Department of Psychiatry, Rotterdam, the Netherlands

^6^ Faculty of Psychology, Padjadjaran University, Bandung, Indonesia

^7^ Health Services Management Department, Guizhou Medical University, Guiyang, China

^8^ Saw Swee Hock School of Public Health, National University of Singapore, Singapore

^9^ Department of Clinical Epidemiology and Biostatistics, Faculty of Medicine Ramathibodi Hospital, Mahidol University, Bangkok, Thailand

^10^ Department of Health Administration and Management, University of Nigeria, Enugu Campus, Enugu State, Nigeria

^11^ Department of Community Medicine and Africa Center of Excellence for Population Health and Policy, Bayero University Kano, Kano state, Nigeria

^12^ Department of Community Medicine, Ahmadu Bello University Teaching Hospital, Zaria, Kaduna State, Nigeria

***Corresponding author**: Montarat Thavorncharoensap

Email: [montarat.tha@mahidol.ac.th](mailto:montarat.tha@mahidol.ac.th)

**SUPPLEMENTARY MATERIAL**

**Supplementary Table 1:** Details of all 34 models

1. **cTTO models with all responses and intercept (Model 1 – 4)**

| **Variable** | **Linear (Model 1)** | | | | | **Tobit (Model 2)** | | | | |
| --- | --- | --- | --- | --- | --- | --- | --- | --- | --- | --- |
|  | **Coefficient** | **SE** | **p-value** | **95%CI** | | **Coefficient** | **SE** | **p-value** | **95%CI** | |
| mo2 | -0.011 | 0.008 | 0.179 | -0.027 | 0.005 | -0.016 | 0.008 | 0.059 | -0.032 | 0.001 |
| mo3 | 0.011 | 0.008 | 0.189 | -0.005 | 0.028 | 0.002 | 0.009 | 0.793 | -0.015 | 0.019 |
| mo4 | 0.214 | 0.009 | <0.001 | 0.196 | 0.232 | 0.204 | 0.009 | <0.001 | 0.185 | 0.222 |
| mo5 | 0.303 | 0.008 | <0.001 | 0.287 | 0.320 | 0.309 | 0.009 | <0.001 | 0.292 | 0.325 |
| sc2 | -0.001 | 0.008 | 0.855 | -0.017 | 0.014 | -0.003 | 0.008 | 0.683 | -0.019 | 0.013 |
| sc3 | 0.057 | 0.009 | <0.001 | 0.039 | 0.074 | 0.055 | 0.009 | <0.001 | 0.037 | 0.073 |
| sc4 | 0.276 | 0.009 | <0.001 | 0.258 | 0.294 | 0.273 | 0.009 | <0.001 | 0.255 | 0.291 |
| sc5 | 0.341 | 0.008 | <0.001 | 0.325 | 0.357 | 0.357 | 0.008 | <0.001 | 0.340 | 0.373 |
| ua2 | 0.015 | 0.008 | 0.066 | -0.001 | 0.032 | 0.014 | 0.009 | 0.112 | -0.003 | 0.030 |
| ua3 | 0.075 | 0.009 | <0.001 | 0.057 | 0.092 | 0.075 | 0.009 | <0.001 | 0.057 | 0.093 |
| ua4 | 0.246 | 0.009 | <0.001 | 0.228 | 0.263 | 0.244 | 0.009 | <0.001 | 0.226 | 0.262 |
| ua5 | 0.277 | 0.008 | <0.001 | 0.261 | 0.293 | 0.292 | 0.008 | <0.001 | 0.276 | 0.309 |
| pd2 | 0.029 | 0.008 | <0.001 | 0.014 | 0.044 | 0.025 | 0.008 | 0.001 | 0.010 | 0.040 |
| pd3 | 0.044 | 0.009 | <0.001 | 0.026 | 0.061 | 0.043 | 0.009 | <0.001 | 0.025 | 0.061 |
| pd4 | 0.305 | 0.008 | <0.001 | 0.289 | 0.320 | 0.308 | 0.008 | <0.001 | 0.292 | 0.324 |
| pd5 | 0.507 | 0.009 | <0.001 | 0.490 | 0.524 | 0.515 | 0.009 | <0.001 | 0.498 | 0.533 |
| ad2 | 0.015 | 0.009 | 0.092 | -0.002 | 0.032 | 0.012 | 0.009 | 0.160 | -0.005 | 0.030 |
| ad3 | 0.062 | 0.010 | <0.001 | 0.043 | 0.081 | 0.056 | 0.010 | <0.001 | 0.036 | 0.075 |
| ad4 | 0.256 | 0.009 | <0.001 | 0.239 | 0.274 | 0.253 | 0.009 | <0.001 | 0.235 | 0.271 |
| ad5 | 0.410 | 0.008 | <0.001 | 0.393 | 0.426 | 0.417 | 0.008 | <0.001 | 0.400 | 0.433 |
| _cons | 0.012 | 0.010 | 0.233 | -0.007 | 0.031 | 0.013 | 0.010 | 0.209 | -0.007 | 0.032 |

| **Variable** | **Linear corrected for heteroskedasticity**  **(Model 3)** | | | | | **Tobit corrected for heteroskedasticity**  **(Model 4)** | | | | |
| --- | --- | --- | --- | --- | --- | --- | --- | --- | --- | --- |
|  | **Coefficient** | **SE** | **p-value** | **95%CI** | | **Coefficient** | **SE** | **p-value** | **95%CI** | |
| mo2 | 0.007 | 0.006 | 0.240 | -0.004 | 0.017 | 0.018 | 0.005 | 0.001 | 0.008 | 0.029 |
| mo3 | 0.024 | 0.009 | 0.007 | 0.007 | 0.042 | 0.051 | 0.009 | <0.001 | 0.033 | 0.068 |
| mo4 | 0.213 | 0.010 | < 0.001 | 0.193 | 0.233 | 0.226 | 0.010 | <0.001 | 0.206 | 0.245 |
| mo5 | 0.319 | 0.008 | <0.001 | 0.302 | 0.335 | 0.296 | 0.008 | <0.001 | 0.279 | 0.312 |
| sc2 | 0.023 | 0.005 | <0.001 | 0.013 | 0.033 | 0.032 | 0.005 | <0.001 | 0.022 | 0.042 |
| sc3 | 0.065 | 0.007 | <0.001 | 0.051 | 0.079 | 0.069 | 0.007 | <0.001 | 0.055 | 0.083 |
| sc4 | 0.247 | 0.010 | <0.001 | 0.229 | 0.266 | 0.245 | 0.009 | <0.001 | 0.226 | 0.263 |
| sc5 | 0.291 | 0.008 | <0.001 | 0.276 | 0.306 | 0.257 | 0.008 | <0.001 | 0.242 | 0.273 |
| ua2 | 0.027 | 0.005 | <0.001 | 0.017 | 0.037 | 0.034 | 0.005 | <0.001 | 0.024 | 0.043 |
| ua3 | 0.081 | 0.008 | <0.001 | 0.066 | 0.096 | 0.080 | 0.007 | <0.001 | 0.065 | 0.095 |
| ua4 | 0.193 | 0.008 | <0.001 | 0.177 | 0.209 | 0.199 | 0.008 | <0.001 | 0.183 | 0.215 |
| ua5 | 0.279 | 0.008 | <0.001 | 0.263 | 0.295 | 0.249 | 0.008 | <0.001 | 0.233 | 0.265 |
| pd2 | 0.032 | 0.005 | <0.001 | 0.023 | 0.041 | 0.038 | 0.004 | <0.001 | 0.029 | 0.046 |
| pd3 | 0.048 | 0.008 | <0.001 | 0.032 | 0.065 | 0.057 | 0.008 | <0.001 | 0.041 | 0.073 |
| pd4 | 0.272 | 0.009 | <0.001 | 0.256 | 0.289 | 0.269 | 0.008 | <0.001 | 0.252 | 0.285 |
| pd5 | 0.483 | 0.010 | <0.001 | 0.465 | 0.502 | 0.465 | 0.009 | <0.001 | 0.447 | 0.484 |
| ad2 | 0.036 | 0.004 | <0.001 | 0.027 | 0.044 | 0.040 | 0.004 | <0.001 | 0.031 | 0.048 |
| ad3 | 0.057 | 0.009 | <0.001 | 0.040 | 0.074 | 0.075 | 0.008 | <0.001 | 0.058 | 0.091 |
| ad4 | 0.211 | 0.008 | <0.001 | 0.196 | 0.227 | 0.216 | 0.008 | <0.001 | 0.201 | 0.231 |
| ad5 | 0.378 | 0.007 | <0.001 | 0.363 | 0.392 | 0.360 | 0.007 | <0.001 | 0.345 | 0.374 |
| _cons | 0.026 | 0.005 | <0.001 | 0.016 | 0.035 | 0.020 | 0.005 | <0.001 | 0.011 | 0.029 |

1. **cTTO models without flagged responses and intercept (Model 5– 8)**

| **Variable** | **Linear (Model 5)** | | | | | **Tobit (Model 6)** | | | | |
| --- | --- | --- | --- | --- | --- | --- | --- | --- | --- | --- |
|  | **Coefficient** | **SE** | **p-value** | **95%CI** | | **Coefficient** | **SE** | **p-value** | **95%CI** | |
| mo2 | -0.010 | 0.008 | 0.201 | -0.026 | 0.006 | **-0.015** | 0.008 | 0.078 | -0.031 | 0.002 |
| mo3 | 0.011 | 0.009 | 0.197 | -0.006 | 0.028 | 0.003 | 0.009 | 0.737 | -0.014 | 0.020 |
| mo4 | 0.219 | 0.009 | <0.001 | 0.200 | 0.237 | 0.210 | 0.010 | <0.001 | 0.191 | 0.228 |
| mo5 | 0.315 | 0.009 | <0.001 | 0.299 | 0.332 | 0.320 | 0.009 | <0.001 | 0.303 | 0.337 |
| sc2 | -0.007 | 0.008 | 0.371 | -0.023 | 0.009 | **-0.009** | 0.008 | 0.259 | -0.026 | 0.007 |
| sc3 | 0.051 | 0.009 | <0.001 | 0.033 | 0.069 | 0.049 | 0.009 | <0.001 | 0.031 | 0.068 |
| sc4 | 0.278 | 0.009 | <0.001 | 0.260 | 0.296 | 0.276 | 0.009 | <0.001 | 0.257 | 0.294 |
| sc5 | 0.335 | 0.008 | <0.001 | 0.319 | 0.352 | 0.349 | 0.008 | <0.001 | 0.333 | 0.366 |
| ua2 | 0.014 | 0.008 | 0.109 | -0.003 | 0.030 | 0.012 | 0.009 | 0.172 | -0.005 | 0.029 |
| ua3 | 0.075 | 0.009 | <0.001 | 0.057 | 0.093 | 0.075 | 0.009 | <0.001 | 0.057 | 0.093 |
| ua4 | 0.241 | 0.009 | <0.001 | 0.223 | 0.258 | 0.239 | 0.009 | <0.001 | 0.221 | 0.257 |
| ua5 | 0.275 | 0.008 | <0.001 | 0.259 | 0.292 | 0.289 | 0.008 | <0.001 | 0.272 | 0.306 |
| pd2 | 0.029 | 0.008 | <0.001 | 0.014 | 0.044 | 0.026 | 0.008 | 0.001 | 0.011 | 0.041 |
| pd3 | 0.044 | 0.009 | <0.001 | 0.026 | 0.062 | 0.043 | 0.009 | <0.001 | 0.024 | 0.061 |
| pd4 | 0.309 | 0.008 | <0.001 | 0.293 | 0.325 | 0.312 | 0.008 | <0.001 | 0.296 | 0.328 |
| pd5 | 0.510 | 0.009 | <0.001 | 0.493 | 0.527 | 0.518 | 0.009 | <0.001 | 0.500 | 0.535 |
| ad2 | 0.018 | 0.009 | 0.038 | 0.001 | 0.035 | 0.016 | 0.009 | 0.068 | -0.001 | 0.034 |
| ad3 | 0.066 | 0.010 | <0.001 | 0.047 | 0.085 | 0.061 | 0.010 | <0.001 | 0.041 | 0.080 |
| ad4 | 0.260 | 0.009 | <0.001 | 0.242 | 0.277 | 0.257 | 0.009 | <0.001 | 0.239 | 0.275 |
| ad5 | 0.410 | 0.008 | <0.001 | 0.393 | 0.426 | 0.416 | 0.009 | <0.001 | 0.400 | 0.433 |
| _cons | 0.012 | 0.010 | 0.203 | -0.007 | 0.032 | 0.013 | 0.010 | 0.180 | -0.006 | 0.033 |

| **Variable** | **Linear corrected for heteroskedasticity (Model 7)** | | | | | **Tobit corrected for heteroskedasticity (Model 8)** | | | | |
| --- | --- | --- | --- | --- | --- | --- | --- | --- | --- | --- |
|  | **Coefficient** | **SE** | **p-value** | **95%CI** | | **Coefficient** | **SE** | **p-value** | **95%CI** | |
| mo2 | 0.011 | 0.006 | 0.058 | <0.001 | 0.021 | 0.021 | 0.005 | <0.001 | 0.010 | 0.031 |
| mo3 | 0.030 | 0.009 | 0.001 | 0.012 | 0.048 | 0.054 | 0.009 | <0.001 | 0.036 | 0.071 |
| mo4 | 0.216 | 0.010 | <0.001 | 0.196 | 0.236 | 0.226 | 0.010 | <0.001 | 0.206 | 0.245 |
| mo5 | 0.325 | 0.008 | <0.001 | 0.308 | 0.341 | 0.303 | 0.009 | <0.001 | 0.286 | 0.319 |
| sc2 | 0.022 | 0.005 | <0.001 | 0.012 | 0.032 | 0.030 | 0.005 | <0.001 | 0.021 | 0.040 |
| sc3 | 0.063 | 0.007 | <0.001 | 0.049 | 0.078 | 0.067 | 0.007 | <0.001 | 0.053 | 0.081 |
| sc4 | 0.251 | 0.010 | <0.001 | 0.232 | 0.270 | 0.248 | 0.009 | <0.001 | 0.229 | 0.266 |
| sc5 | 0.285 | 0.008 | <0.001 | 0.270 | 0.301 | 0.255 | 0.008 | <0.001 | 0.240 | 0.271 |
| ua2 | 0.028 | 0.005 | <0.001 | 0.018 | 0.038 | 0.034 | 0.005 | <0.001 | 0.024 | 0.043 |
| ua3 | 0.080 | 0.008 | <0.001 | 0.065 | 0.095 | 0.079 | 0.008 | <0.001 | 0.064 | 0.094 |
| ua4 | 0.193 | 0.008 | <0.001 | 0.176 | 0.209 | 0.198 | 0.008 | <0.001 | 0.183 | 0.214 |
| ua5 | 0.278 | 0.008 | <0.001 | 0.262 | 0.294 | 0.250 | 0.008 | <0.001 | 0.234 | 0.266 |
| pd2 | 0.035 | 0.004 | <0.001 | 0.026 | 0.044 | 0.040 | 0.004 | <0.001 | 0.031 | 0.048 |
| pd3 | 0.051 | 0.009 | <0.001 | 0.035 | 0.068 | 0.059 | 0.008 | <0.001 | 0.043 | 0.076 |
| pd4 | 0.277 | 0.009 | <0.001 | 0.260 | 0.294 | 0.274 | 0.009 | <0.001 | 0.257 | 0.291 |
| pd5 | 0.486 | 0.010 | <0.001 | 0.467 | 0.505 | 0.470 | 0.010 | <0.001 | 0.451 | 0.489 |
| ad2 | 0.038 | 0.004 | <0.001 | 0.030 | 0.047 | 0.042 | 0.004 | <0.001 | 0.033 | 0.050 |
| ad3 | 0.060 | 0.009 | <0.001 | 0.043 | 0.077 | 0.076 | 0.008 | <0.001 | 0.060 | 0.093 |
| ad4 | 0.213 | 0.008 | <0.001 | 0.197 | 0.229 | 0.218 | 0.008 | <0.001 | 0.202 | 0.233 |
| ad5 | 0.377 | 0.007 | <0.001 | 0.362 | 0.391 | 0.361 | 0.007 | <0.001 | 0.347 | 0.375 |
| _cons | 0.024 | 0.005 | <0.001 | 0.015 | 0.034 | 0.019 | 0.005 | <0.001 | 0.010 | 0.028 |

1. **cTTO models with all responses and intercept constrained (Model 9-12)**

| **Variable** | **Linear (Model 9)** | | | | | **Tobit (Model 10)** | | | | |
| --- | --- | --- | --- | --- | --- | --- | --- | --- | --- | --- |
|  | **Coefficient** | **SE** | **p-value** | **95%CI** | | **Coefficient** | **SE** | **p-**  **value** | **95%CI** | |
| mo2 | **-0.008** | 0.008 | 0.294 | -0.023 | 0.007 | **-0.013** | 0.008 | 0.109 | -0.028 | 0.003 |
| mo3 | 0.013 | 0.008 | 0.130 | -0.004 | 0.029 | 0.004 | 0.008 | 0.646 | -0.013 | 0.021 |
| mo4 | 0.216 | 0.009 | <0.001 | 0.198 | 0.234 | 0.206 | 0.009 | <0.001 | 0.188 | 0.224 |
| mo5 | 0.304 | 0.008 | <0.001 | 0.288 | 0.321 | 0.309 | 0.009 | <0.001 | 0.293 | 0.326 |
| sc2 | 0.002 | 0.007 | 0.772 | -0.012 | 0.017 | 0.001 | 0.008 | 0.945 | -0.014 | 0.015 |
| sc3 | 0.057 | 0.009 | <0.001 | 0.040 | 0.075 | 0.056 | 0.009 | <0.001 | 0.038 | 0.074 |
| sc4 | 0.277 | 0.009 | <0.001 | 0.260 | 0.295 | 0.274 | 0.009 | <0.001 | 0.257 | 0.292 |
| sc5 | 0.342 | 0.008 | <0.001 | 0.326 | 0.358 | 0.358 | 0.008 | <0.001 | 0.342 | 0.374 |
| ua2 | 0.019 | 0.008 | 0.015 | 0.004 | 0.034 | 0.017 | 0.008 | 0.029 | 0.002 | 0.033 |
| ua3 | 0.078 | 0.009 | <0.001 | 0.061 | 0.095 | 0.078 | 0.009 | <0.001 | 0.061 | 0.095 |
| ua4 | 0.248 | 0.009 | <0.001 | 0.232 | 0.265 | 0.247 | 0.009 | <0.001 | 0.230 | 0.264 |
| ua5 | 0.279 | 0.008 | <0.001 | 0.263 | 0.295 | 0.294 | 0.008 | <0.001 | 0.278 | 0.310 |
| pd2 | 0.032 | 0.007 | <0.001 | 0.019 | 0.046 | 0.029 | 0.007 | <0.001 | 0.015 | 0.043 |
| pd3 | 0.044 | 0.009 | <0.001 | 0.026 | 0.062 | 0.043 | 0.009 | <0.001 | 0.025 | 0.061 |
| pd4 | 0.304 | 0.008 | <0.001 | 0.288 | 0.320 | 0.308 | 0.008 | <0.001 | 0.291 | 0.324 |
| pd5 | 0.510 | 0.008 | <0.001 | 0.493 | 0.526 | 0.518 | 0.009 | <0.001 | 0.501 | 0.535 |
| ad2 | 0.020 | 0.008 | 0.011 | 0.005 | 0.035 | 0.018 | 0.008 | 0.024 | 0.002 | 0.033 |
| ad3 | 0.066 | 0.009 | <0.001 | 0.048 | 0.084 | 0.060 | 0.009 | <0.001 | 0.041 | 0.078 |
| ad4 | 0.259 | 0.008 | <0.001 | 0.243 | 0.276 | 0.257 | 0.009 | <0.001 | 0.240 | 0.274 |
| ad5 | 0.412 | 0.008 | <0.001 | 0.397 | 0.428 | 0.420 | 0.008 | <0.001 | 0.404 | 0.436 |
| _cons |  |  |  |  |  |  |  |  |  |  |

| **Variable** | **Linear corrected for heteroskedasticity**  **(Model 11)** | | | | | **Tobit corrected for heteroskedasticity**  **(Model 12)** | | | | |
| --- | --- | --- | --- | --- | --- | --- | --- | --- | --- | --- |
|  | **Coefficient** | **SE** | **p-value** | **95%CI** | | **Coefficient** | **SE** | **p-value** | **95%CI** | |
| mo2 | 0.022 | 0.005 | <0.001 | 0.012 | 0.031 | 0.031 | 0.005 | <0.001 | 0.022 | 0.040 |
| mo3 | 0.031 | 0.009 | 0.001 | 0.013 | 0.049 | 0.056 | 0.009 | <0.001 | 0.039 | 0.074 |
| mo4 | 0.219 | 0.010 | <0.001 | 0.200 | 0.239 | 0.231 | 0.010 | <0.001 | 0.211 | 0.250 |
| mo5 | 0.319 | 0.008 | <0.001 | 0.302 | 0.335 | 0.296 | 0.008 | <0.001 | 0.279 | 0.312 |
| sc2 | 0.039 | 0.004 | <0.001 | 0.031 | 0.047 | 0.045 | 0.004 | <0.001 | 0.037 | 0.053 |
| sc3 | 0.064 | 0.007 | <0.001 | 0.049 | 0.078 | 0.068 | 0.007 | <0.001 | 0.054 | 0.081 |
| sc4 | 0.252 | 0.010 | <0.001 | 0.233 | 0.270 | 0.248 | 0.009 | <0.001 | 0.229 | 0.266 |
| sc5 | 0.291 | 0.008 | <0.001 | 0.275 | 0.306 | 0.256 | 0.008 | <0.001 | 0.241 | 0.272 |
| ua2 | 0.045 | 0.004 | <0.001 | 0.037 | 0.052 | 0.047 | 0.004 | <0.001 | 0.040 | 0.055 |
| ua3 | 0.091 | 0.008 | <0.001 | 0.076 | 0.106 | 0.088 | 0.007 | <0.001 | 0.073 | 0.102 |
| ua4 | 0.203 | 0.008 | <0.001 | 0.188 | 0.219 | 0.207 | 0.008 | <0.001 | 0.192 | 0.223 |
| ua5 | 0.285 | 0.008 | <0.001 | 0.269 | 0.300 | 0.252 | 0.008 | <0.001 | 0.236 | 0.268 |
| pd2 | 0.046 | 0.004 | <0.001 | 0.038 | 0.053 | 0.049 | 0.004 | <0.001 | 0.042 | 0.056 |
| pd3 | 0.047 | 0.008 | <0.001 | 0.030 | 0.063 | 0.056 | 0.008 | <0.001 | 0.040 | 0.072 |
| pd4 | 0.266 | 0.009 | <0.001 | 0.250 | 0.283 | 0.264 | 0.008 | <0.001 | 0.247 | 0.280 |
| pd5 | 0.496 | 0.009 | <0.001 | 0.478 | 0.514 | 0.475 | 0.009 | <0.001 | 0.457 | 0.493 |
| ad2 | 0.050 | 0.004 | <0.001 | 0.043 | 0.057 | 0.050 | 0.003 | <0.001 | 0.044 | 0.057 |
| ad3 | 0.068 | 0.008 | <0.001 | 0.052 | 0.084 | 0.084 | 0.008 | <0.001 | 0.068 | 0.100 |
| ad4 | 0.216 | 0.008 | <0.001 | 0.201 | 0.232 | 0.220 | 0.008 | <0.001 | 0.205 | 0.235 |
| ad5 | 0.382 | 0.007 | <0.001 | 0.368 | 0.396 | 0.363 | 0.007 | <0.001 | 0.349 | 0.377 |
| _cons |  |  |  |  |  |  |  |  |  |  |

1. **cTTO models without flagged responses and intercept constrained (Model 13-16)**

| **Variable** | **Linear (Model 13)** | | | | | **Tobit (Model 14)** | | | | |
| --- | --- | --- | --- | --- | --- | --- | --- | --- | --- | --- |
|  | **Coefficient** | **SE** | **p-**  **value** | **95%CI** | | **Coefficient** | **SE** | **p-**  **value** | **95%CI** | |
| mo2 | **-0.007** | 0.008 | 0.343 | -0.023 | 0.008 | **-0.011** | 0.008 | 0.152 | -0.027 | 0.004 |
| mo3 | 0.013 | 0.008 | 0.132 | -0.004 | 0.029 | 0.005 | 0.009 | 0.583 | -0.012 | 0.022 |
| mo4 | 0.221 | 0.009 | <0.001 | 0.203 | 0.239 | 0.212 | 0.009 | <0.001 | 0.194 | 0.230 |
| mo5 | 0.316 | 0.009 | <0.001 | 0.299 | 0.333 | 0.321 | 0.009 | <0.001 | 0.304 | 0.338 |
| sc2 | **-0.003** | 0.008 | 0.656 | -0.018 | 0.011 | **-0.005** | 0.008 | 0.502 | -0.020 | 0.010 |
| sc3 | 0.052 | 0.009 | <0.001 | 0.035 | 0.070 | 0.051 | 0.009 | <0.001 | 0.032 | 0.069 |
| sc4 | 0.279 | 0.009 | <0.001 | 0.262 | 0.297 | 0.277 | 0.009 | <0.001 | 0.259 | 0.295 |
| sc5 | 0.337 | 0.008 | <0.001 | 0.321 | 0.353 | 0.351 | 0.008 | <0.001 | 0.334 | 0.367 |
| ua2 | 0.017 | 0.008 | 0.027 | 0.002 | 0.033 | 0.016 | 0.008 | 0.047 | <0.001 | 0.032 |
| ua3 | 0.078 | 0.009 | <0.001 | 0.062 | 0.095 | 0.079 | 0.009 | <0.001 | 0.062 | 0.096 |
| ua4 | 0.244 | 0.009 | <0.001 | 0.227 | 0.261 | 0.242 | 0.009 | <0.001 | 0.225 | 0.260 |
| ua5 | 0.277 | 0.008 | <0.001 | 0.261 | 0.293 | 0.291 | 0.008 | <0.001 | 0.275 | 0.308 |
| pd2 | 0.033 | 0.007 | <0.001 | 0.019 | 0.047 | 0.030 | 0.007 | <0.001 | 0.016 | 0.044 |
| pd3 | 0.044 | 0.009 | <0.001 | 0.026 | 0.062 | 0.043 | 0.009 | <0.001 | 0.025 | 0.062 |
| pd4 | 0.309 | 0.008 | <0.001 | 0.293 | 0.325 | 0.312 | 0.008 | <0.001 | 0.295 | 0.328 |
| pd5 | 0.513 | 0.009 | <0.001 | 0.496 | 0.530 | 0.520 | 0.009 | <0.001 | 0.503 | 0.538 |
| ad2 | 0.023 | 0.008 | 0.002 | 0.008 | 0.039 | 0.022 | 0.008 | 0.005 | 0.007 | 0.037 |
| ad3 | 0.070 | 0.009 | <0.001 | 0.052 | 0.088 | 0.065 | 0.009 | <0.001 | 0.046 | 0.084 |
| ad4 | 0.263 | 0.009 | <0.001 | 0.246 | 0.280 | 0.261 | 0.009 | <0.001 | 0.244 | 0.278 |
| ad5 | 0.413 | 0.008 | <0.001 | 0.397 | 0.428 | 0.419 | 0.008 | <0.001 | 0.403 | 0.435 |
| _cons |  |  |  |  |  |  |  |  |  |  |

| **Variable** | **Linear corrected for heteroskedasticity**  **(Model 15)** | | | | | **Tobit corrected for heteroskedasticity**  **(Model 16)** | | | | |
| --- | --- | --- | --- | --- | --- | --- | --- | --- | --- | --- |
|  | **Coefficient** | **SE** | **P**  **-value** | **95%CI** | | **Coefficient** | **SE** | **p-**  **value** | **95%CI** | |
| mo2 | 0.025 | 0.005 | <0.001 | 0.016 | 0.035 | 0.033 | 0.004 | <0.001 | 0.024 | 0.042 |
| mo3 | 0.037 | 0.009 | <0.001 | 0.019 | 0.055 | 0.059 | 0.009 | <0.001 | 0.042 | 0.077 |
| mo4 | 0.222 | 0.010 | <0.001 | 0.202 | 0.242 | 0.230 | 0.010 | <0.001 | 0.211 | 0.250 |
| mo5 | 0.324 | 0.009 | <0.001 | 0.308 | 0.341 | 0.302 | 0.009 | <0.001 | 0.286 | 0.319 |
| sc2 | 0.038 | 0.004 | <0.001 | 0.030 | 0.046 | 0.043 | 0.004 | <0.001 | 0.036 | 0.050 |
| sc3 | 0.062 | 0.007 | <0.001 | 0.048 | 0.077 | 0.066 | 0.007 | <0.001 | 0.052 | 0.081 |
| sc4 | 0.255 | 0.010 | <0.001 | 0.237 | 0.274 | 0.251 | 0.009 | <0.001 | 0.232 | 0.269 |
| sc5 | 0.285 | 0.008 | <0.001 | 0.269 | 0.300 | 0.254 | 0.008 | <0.001 | 0.239 | 0.270 |
| ua2 | 0.044 | 0.004 | <0.001 | 0.037 | 0.052 | 0.047 | 0.004 | <0.001 | 0.039 | 0.054 |
| ua3 | 0.089 | 0.008 | <0.001 | 0.074 | 0.104 | 0.086 | 0.007 | <0.001 | 0.071 | 0.100 |
| ua4 | 0.203 | 0.008 | <0.001 | 0.187 | 0.218 | 0.206 | 0.008 | <0.001 | 0.191 | 0.222 |
| ua5 | 0.283 | 0.008 | <0.001 | 0.267 | 0.299 | 0.253 | 0.008 | <0.001 | 0.237 | 0.269 |
| pd2 | 0.048 | 0.004 | <0.001 | 0.040 | 0.055 | 0.050 | 0.003 | <0.001 | 0.043 | 0.057 |
| pd3 | 0.049 | 0.009 | <0.001 | 0.033 | 0.066 | 0.058 | 0.008 | <0.001 | 0.042 | 0.074 |
| pd4 | 0.271 | 0.009 | <0.001 | 0.254 | 0.288 | 0.269 | 0.009 | <0.001 | 0.252 | 0.286 |
| pd5 | 0.498 | 0.009 | <0.001 | 0.480 | 0.516 | 0.480 | 0.009 | <0.001 | 0.461 | 0.498 |
| ad2 | 0.051 | 0.004 | <0.001 | 0.044 | 0.058 | 0.052 | 0.003 | <0.001 | 0.045 | 0.059 |
| ad3 | 0.070 | 0.008 | <0.001 | 0.053 | 0.086 | 0.084 | 0.008 | <0.001 | 0.068 | 0.101 |
| ad4 | 0.217 | 0.008 | <0.001 | 0.202 | 0.233 | 0.221 | 0.008 | <0.001 | 0.205 | 0.236 |
| ad5 | 0.381 | 0.007 | <0.001 | 0.366 | 0.395 | 0.364 | 0.007 | <0.001 | 0.349 | 0.378 |
| _cons |  |  |  |  |  |  |  |  |  |  |

1. **DCE models (Model 17-18)**

| **Variable** | **DCE Conditional Logit Model (Model 17)** | | | | | **DCE Mixed Logit Model (Model 18)** | | | | |
| --- | --- | --- | --- | --- | --- | --- | --- | --- | --- | --- |
|  | **Coefficient** | **SE** | **p-**  **value** | **95%CI** | | **Coefficient** | **SE** | **MCSE** | **95%CI** | |
| mo2 | 0.327 | 0.047 | <0.001 | 0.235 | 0.418 | 0.507 | 0.064 | 0.019 | 0.374 | 0.622 |
| mo3 | 0.811 | 0.049 | <0.001 | 0.715 | 0.907 | 1.257 | 0.068 | 0.014 | 1.112 | 1.387 |
| mo4 | 1.379 | 0.065 | <0.001 | 1.251 | 1.507 | 2.308 | 0.106 | 0.025 | 2.124 | 2.531 |
| mo5 | 2.340 | 0.085 | <0.001 | 2.174 | 2.506 | 3.869 | 0.172 | 0.040 | 3.549 | 4.215 |
| sc2 | 0.183 | 0.045 | <0.001 | 0.094 | 0.271 | 0.304 | 0.065 | 0.014 | 0.185 | 0.421 |
| sc3 | 0.619 | 0.046 | <0.001 | 0.528 | 0.710 | 0.990 | 0.069 | 0.011 | 0.853 | 1.120 |
| sc4 | 1.090 | 0.057 | <0.001 | 0.977 | 1.202 | 1.852 | 0.098 | 0.018 | 1.663 | 2.033 |
| sc5 | 1.716 | 0.066 | <0.001 | 1.586 | 1.846 | 2.873 | 0.114 | 0.022 | 2.665 | 3.104 |
| ua2 | 0.256 | 0.045 | <0.001 | 0.168 | 0.344 | 0.391 | 0.085 | 0.021 | 0.240 | 0.589 |
| ua3 | 0.636 | 0.046 | <0.001 | 0.546 | 0.725 | 0.990 | 0.101 | 0.025 | 0.801 | 1.204 |
| ua4 | 1.272 | 0.055 | <0.001 | 1.165 | 1.380 | 2.025 | 0.118 | 0.029 | 1.810 | 2.250 |
| ua5 | 1.910 | 0.066 | <0.001 | 1.780 | 2.040 | 3.131 | 0.154 | 0.037 | 2.857 | 3.443 |
| pd2 | 0.328 | 0.047 | <0.001 | 0.236 | 0.420 | 0.516 | 0.068 | 0.018 | 0.371 | 0.637 |
| pd3 | 0.953 | 0.050 | <0.001 | 0.856 | 1.051 | 1.537 | 0.078 | 0.018 | 1.387 | 1.672 |
| pd4 | 1.960 | 0.076 | <0.001 | 1.812 | 2.109 | 3.282 | 0.138 | 0.027 | 3.021 | 3.561 |
| pd5 | 3.101 | 0.096 | <0.001 | 2.913 | 3.289 | 5.354 | 0.196 | 0.037 | 4.992 | 5.748 |
| ad2 | 0.176 | 0.046 | <0.001 | 0.086 | 0.265 | 0.363 | 0.059 | 0.016 | 0.255 | 0.497 |
| ad3 | 0.593 | 0.049 | <0.001 | 0.498 | 0.688 | 1.041 | 0.064 | 0.014 | 0.917 | 1.177 |
| ad4 | 1.405 | 0.068 | <0.001 | 1.272 | 1.538 | 2.378 | 0.108 | 0.025 | 2.190 | 2.607 |
| ad5 | 2.315 | 0.082 | <0.001 | 2.154 | 2.476 | 3.941 | 0.165 | 0.036 | 3.634 | 4.271 |
| _cons |  |  |  |  |  |  |  |  |  |  |

1. **Hybrid models with all responses and intercept (Model 19-22)**

| **Variable** | **Linear (Model 19)** | | | | | **Tobit (Model 20)** | | | | |
| --- | --- | --- | --- | --- | --- | --- | --- | --- | --- | --- |
|  | **Coefficient** | **SE** | **p-value** | **95%CI** | | **Coefficient** | **SE** | **p-value** | **95%CI** | |
| mo2 | 0.019 | 0.006 | 0.001 | 0.008 | 0.031 | 0.015 | 0.006 | 0.013 | 0.003 | 0.027 |
| mo3 | 0.084 | 0.006 | <0.001 | 0.072 | 0.096 | 0.080 | 0.006 | <0.001 | 0.068 | 0.092 |
| mo4 | 0.215 | 0.006 | <0.001 | 0.203 | 0.227 | 0.212 | 0.006 | <0.001 | 0.200 | 0.225 |
| mo5 | 0.348 | 0.006 | <0.001 | 0.336 | 0.361 | 0.355 | 0.006 | <0.001 | 0.342 | 0.367 |
| sc2 | 0.008 | 0.006 | 0.142 | -0.003 | 0.019 | 0.005 | 0.006 | 0.366 | -0.006 | 0.017 |
| sc3 | 0.077 | 0.006 | <0.001 | 0.066 | 0.089 | 0.076 | 0.006 | <0.001 | 0.064 | 0.088 |
| sc4 | 0.203 | 0.006 | <0.001 | 0.191 | 0.215 | 0.202 | 0.006 | <0.001 | 0.190 | 0.215 |
| sc5 | 0.289 | 0.006 | <0.001 | 0.277 | 0.300 | 0.299 | 0.006 | <0.001 | 0.287 | 0.311 |
| ua2 | 0.012 | 0.006 | 0.030 | 0.001 | 0.023 | 0.010 | 0.006 | 0.095 | -0.002 | 0.021 |
| ua3 | 0.079 | 0.006 | <0.001 | 0.068 | 0.090 | 0.079 | 0.006 | <0.001 | 0.067 | 0.091 |
| ua4 | 0.207 | 0.006 | <0.001 | 0.195 | 0.219 | 0.208 | 0.006 | <0.001 | 0.196 | 0.221 |
| ua5 | 0.300 | 0.006 | <0.001 | 0.289 | 0.311 | 0.310 | 0.006 | <0.001 | 0.298 | 0.321 |
| pd2 | 0.042 | 0.005 | <0.001 | 0.031 | 0.053 | 0.040 | 0.006 | <0.001 | 0.029 | 0.051 |
| pd3 | 0.116 | 0.006 | <0.001 | 0.104 | 0.128 | 0.116 | 0.006 | <0.001 | 0.104 | 0.128 |
| pd4 | 0.321 | 0.006 | <0.001 | 0.309 | 0.333 | 0.324 | 0.006 | <0.001 | 0.312 | 0.336 |
| pd5 | 0.519 | 0.006 | <0.001 | 0.507 | 0.532 | 0.532 | 0.007 | <0.001 | 0.519 | 0.545 |
| ad2 | 0.014 | 0.006 | 0.018 | 0.002 | 0.025 | 0.012 | 0.006 | 0.050 | <0.001 | 0.024 |
| ad3 | 0.076 | 0.006 | <0.001 | 0.064 | 0.088 | 0.073 | 0.006 | <0.001 | 0.060 | 0.085 |
| ad4 | 0.232 | 0.006 | <0.001 | 0.220 | 0.244 | 0.232 | 0.006 | <0.001 | 0.220 | 0.245 |
| ad5 | 0.394 | 0.006 | <0.001 | 0.382 | 0.406 | 0.402 | 0.006 | <0.001 | 0.390 | 0.415 |
| _cons | -0.003 | 0.003 | 0.268 | -0.009 | 0.003 | -0.004 | 0.003 | 0.187 | -0.011 | 0.002 |

| **Variable** | **Linear corrected for heteroskedasticity**  **(Model 21)** | | | | | **Tobit corrected for heteroskedasticity**  **(Model 22)** | | | | |
| --- | --- | --- | --- | --- | --- | --- | --- | --- | --- | --- |
|  | **Coefficient** | **SE** | **p-value** | **95%CI** | | **Coefficient** | **SE** | **p-value** | **95%CI** | |
| mo2 | 0.025 | 0.004 | <0.001 | 0.017 | 0.032 | **0.026** | **0.004** | **<0.001** | **0.019** | **0.033** |
| mo3 | 0.088 | 0.005 | <0.001 | 0.078 | 0.098 | **0.087** | **0.005** | **<0.001** | **0.077** | **0.097** |
| mo4 | 0.198 | 0.005 | <0.001 | 0.187 | 0.208 | **0.195** | **0.005** | **<0.001** | **0.185** | **0.206** |
| mo5 | 0.337 | 0.005 | <0.001 | 0.327 | 0.347 | **0.332** | **0.005** | **<0.001** | **0.322** | **0.343** |
| sc2 | 0.030 | 0.004 | <0.001 | 0.022 | 0.037 | **0.032** | **0.003** | **<0.001** | **0.025** | **0.039** |
| sc3 | 0.084 | 0.005 | <0.001 | 0.075 | 0.093 | **0.084** | **0.005** | **<0.001** | **0.076** | **0.093** |
| sc4 | 0.184 | 0.005 | <0.001 | 0.174 | 0.195 | **0.182** | **0.005** | **<0.001** | **0.172** | **0.192** |
| sc5 | 0.267 | 0.005 | <0.001 | 0.258 | 0.277 | **0.265** | **0.005** | **<0.001** | **0.255** | **0.274** |
| ua2 | 0.030 | 0.004 | <0.001 | 0.023 | 0.037 | **0.031** | **0.003** | **<0.001** | **0.024** | **0.038** |
| ua3 | 0.078 | 0.005 | <0.001 | 0.068 | 0.087 | **0.079** | **0.004** | **<0.001** | **0.070** | **0.087** |
| ua4 | 0.189 | 0.005 | <0.001 | 0.180 | 0.199 | **0.189** | **0.005** | **<0.001** | **0.179** | **0.198** |
| ua5 | 0.284 | 0.005 | <0.001 | 0.275 | 0.294 | **0.283** | **0.005** | **<0.001** | **0.273** | **0.293** |
| pd2 | 0.043 | 0.003 | <0.001 | 0.036 | 0.049 | **0.044** | **0.003** | **<0.001** | **0.037** | **0.050** |
| pd3 | 0.112 | 0.005 | <0.001 | 0.103 | 0.122 | **0.112** | **0.005** | **<0.001** | **0.103** | **0.121** |
| pd4 | 0.286 | 0.005 | <0.001 | 0.276 | 0.297 | **0.285** | **0.005** | **<0.001** | **0.275** | **0.295** |
| pd5 | 0.481 | 0.006 | <0.001 | 0.470 | 0.493 | **0.477** | **0.006** | **<0.001** | **0.465** | **0.490** |
| ad2 | 0.038 | 0.003 | <0.001 | 0.031 | 0.045 | **0.039** | **0.003** | **<0.001** | **0.033** | **0.045** |
| ad3 | 0.084 | 0.005 | <0.001 | 0.075 | 0.094 | **0.086** | **0.005** | **<0.001** | **0.077** | **0.095** |
| ad4 | 0.207 | 0.005 | <0.001 | 0.197 | 0.217 | **0.206** | **0.005** | **<0.001** | **0.196** | **0.215** |
| ad5 | 0.367 | 0.005 | <0.001 | 0.358 | 0.377 | **0.365** | **0.005** | **<0.001** | **0.355** | **0.374** |
| _cons | 0.009 | 0.002 | <0.001 | 0.004 | 0.013 | **0.010** | **0.002** | **<0.001** | **0.005** | **0.014** |

1. **Hybrid models without flagged responses and intercept (Model 23-26)**

| **Variable** | **Linear (Model 23)** | | | | | **Tobit (Model 24)** | | | | |
| --- | --- | --- | --- | --- | --- | --- | --- | --- | --- | --- |
|  | **Coefficient** | **SE** | **p-value** | **95%CI** | | **Coefficient** | **SE** | **p-value** | **95%CI** | |
| mo2 | 0.022 | 0.006 | <0.001 | 0.010 | 0.033 | 0.018 | 0.006 | 0.003 | 0.006 | 0.030 |
| mo3 | 0.087 | 0.006 | <0.001 | 0.075 | 0.099 | 0.083 | 0.006 | <0.001 | 0.071 | 0.096 |
| mo4 | 0.218 | 0.006 | <0.001 | 0.206 | 0.230 | 0.216 | 0.006 | <0.001 | 0.204 | 0.229 |
| mo5 | 0.354 | 0.006 | <0.001 | 0.342 | 0.366 | 0.360 | 0.006 | <0.001 | 0.348 | 0.373 |
| sc2 | 0.007 | 0.006 | 0.238 | -0.004 | 0.018 | 0.004 | 0.006 | 0.516 | -0.008 | 0.015 |
| sc3 | 0.077 | 0.006 | <0.001 | 0.065 | 0.088 | 0.075 | 0.006 | <0.001 | 0.063 | 0.088 |
| sc4 | 0.204 | 0.006 | <0.001 | 0.192 | 0.216 | 0.204 | 0.006 | <0.001 | 0.192 | 0.216 |
| sc5 | 0.286 | 0.006 | <0.001 | 0.275 | 0.298 | 0.295 | 0.006 | <0.001 | 0.283 | 0.307 |
| ua2 | 0.012 | 0.006 | 0.028 | 0.001 | 0.024 | 0.010 | 0.006 | 0.082 | -0.001 | 0.022 |
| ua3 | 0.079 | 0.006 | <0.001 | 0.068 | 0.091 | 0.079 | 0.006 | <0.001 | 0.068 | 0.091 |
| ua4 | 0.206 | 0.006 | <0.001 | 0.195 | 0.218 | 0.208 | 0.006 | <0.001 | 0.195 | 0.220 |
| ua5 | 0.300 | 0.006 | <0.001 | 0.288 | 0.311 | 0.309 | 0.006 | <0.001 | 0.297 | 0.320 |
| pd2 | 0.043 | 0.005 | <0.001 | 0.033 | 0.054 | 0.041 | 0.006 | <0.001 | 0.030 | 0.052 |
| pd3 | 0.119 | 0.006 | <0.001 | 0.107 | 0.130 | 0.118 | 0.006 | <0.001 | 0.106 | 0.131 |
| pd4 | 0.325 | 0.006 | <0.001 | 0.313 | 0.336 | 0.327 | 0.006 | <0.001 | 0.315 | 0.340 |
| pd5 | 0.521 | 0.006 | <0.001 | 0.508 | 0.534 | 0.533 | 0.007 | <0.001 | 0.520 | 0.546 |
| ad2 | 0.017 | 0.006 | 0.004 | 0.005 | 0.028 | 0.015 | 0.006 | 0.013 | 0.003 | 0.027 |
| ad3 | 0.079 | 0.006 | <0.001 | 0.068 | 0.091 | 0.077 | 0.006 | <0.001 | 0.064 | 0.089 |
| ad4 | 0.235 | 0.006 | <0.001 | 0.223 | 0.247 | 0.235 | 0.006 | <0.001 | 0.223 | 0.248 |
| ad5 | 0.394 | 0.006 | <0.001 | 0.383 | 0.406 | 0.402 | 0.006 | <0.001 | 0.390 | 0.414 |
| _cons | -0.003 | 0.003 | 0.253 | -0.009 | 0.003 | -0.004 | 0.003 | 0.182 | -0.010 | 0.002 |

| **Variable** | **Linear corrected for heteroskedasticity**  **(Model 25)** | | | | | **Tobit corrected for heteroskedasticity**  **(Model 26)** | | | | |
| --- | --- | --- | --- | --- | --- | --- | --- | --- | --- | --- |
|  | **Coefficient** | **SE** | **p-value** | **95%CI** | | **Coefficient** | **SE** | **p-value** | **95%CI** | |
| mo2 | 0.027 | 0.004 | <0.001 | 0.020 | 0.035 | **0.028** | **0.004** | **<0.001** | **0.021** | **0.036** |
| mo3 | 0.091 | 0.005 | <0.001 | 0.081 | 0.101 | **0.090** | **0.005** | **<0.001** | **0.080** | **0.100** |
| mo4 | 0.200 | 0.005 | <0.001 | 0.190 | 0.211 | **0.197** | **0.005** | **<0.001** | **0.187** | **0.208** |
| mo5 | 0.340 | 0.005 | <0.001 | 0.329 | 0.350 | **0.335** | **0.005** | **<0.001** | **0.325** | **0.346** |
| sc2 | 0.029 | 0.004 | <0.001 | 0.022 | 0.036 | **0.031** | **0.003** | **<0.001** | **0.025** | **0.038** |
| sc3 | 0.084 | 0.005 | <0.001 | 0.075 | 0.093 | **0.084** | **0.005** | **<0.001** | **0.075** | **0.093** |
| sc4 | 0.185 | 0.005 | <0.001 | 0.175 | 0.195 | **0.183** | **0.005** | **<0.001** | **0.173** | **0.193** |
| sc5 | 0.265 | 0.005 | <0.001 | 0.256 | 0.275 | **0.263** | **0.005** | **<0.001** | **0.254** | **0.272** |
| ua2 | 0.031 | 0.004 | <0.001 | 0.024 | 0.038 | **0.031** | **0.003** | **<0.001** | **0.025** | **0.038** |
| ua3 | 0.077 | 0.005 | <0.001 | 0.068 | 0.086 | **0.078** | **0.004** | **<0.001** | **0.070** | **0.086** |
| ua4 | 0.189 | 0.005 | <0.001 | 0.180 | 0.199 | **0.188** | **0.005** | **<0.001** | **0.179** | **0.198** |
| ua5 | 0.283 | 0.005 | <0.001 | 0.274 | 0.293 | **0.282** | **0.005** | **<0.001** | **0.273** | **0.292** |
| pd2 | 0.044 | 0.003 | <0.001 | 0.038 | 0.051 | **0.045** | **0.003** | **<0.001** | **0.039** | **0.051** |
| pd3 | 0.115 | 0.005 | <0.001 | 0.105 | 0.125 | **0.115** | **0.004** | **<0.001** | **0.106** | **0.123** |
| pd4 | 0.289 | 0.005 | <0.001 | 0.279 | 0.300 | **0.288** | **0.005** | **<0.001** | **0.278** | **0.298** |
| pd5 | 0.482 | 0.006 | <0.001 | 0.471 | 0.494 | **0.479** | **0.006** | **<0.001** | **0.467** | **0.491** |
| ad2 | 0.040 | 0.003 | <0.001 | 0.033 | 0.046 | **0.041** | **0.003** | **<0.001** | **0.035** | **0.047** |
| ad3 | 0.086 | 0.005 | <0.001 | 0.076 | 0.096 | **0.087** | **0.005** | **<0.001** | **0.078** | **0.097** |
| ad4 | 0.209 | 0.005 | <0.001 | 0.199 | 0.219 | **0.207** | **0.005** | **<0.001** | **0.198** | **0.217** |
| ad5 | 0.367 | 0.005 | <0.001 | 0.357 | 0.377 | **0.365** | **0.005** | **<0.001** | **0.355** | **0.375** |
| _cons | 0.008 | 0.002 | <0.001 | 0.004 | 0.013 | **0.009** | **0.002** | **<0.001** | **0.005** | **0.014** |

1. **Hybrid models with all responses and intercept constrained (Model 27-30)**

| **Variable** | **Linear (Model 27)** | | | | | **Tobit (Model 28)** | | | | |
| --- | --- | --- | --- | --- | --- | --- | --- | --- | --- | --- |
|  | **Coefficient** | **SE** | **p-value** | **95%CI** | | **Coefficient** | **SE** | **p-value** | **95%CI** | |
| mo2 | 0.019 | 0.006 | 0.001 | 0.008 | 0.031 | 0.015 | 0.006 | 0.017 | 0.003 | 0.027 |
| mo3 | 0.084 | 0.006 | <0.001 | 0.072 | 0.096 | 0.080 | 0.006 | <0.001 | 0.067 | 0.092 |
| mo4 | 0.214 | 0.006 | <0.001 | 0.202 | 0.226 | 0.211 | 0.006 | <0.001 | 0.199 | 0.224 |
| mo5 | 0.348 | 0.006 | <0.001 | 0.336 | 0.360 | 0.354 | 0.006 | <0.001 | 0.341 | 0.366 |
| sc2 | 0.008 | 0.006 | 0.174 | -0.003 | 0.019 | 0.004 | 0.006 | 0.446 | -0.007 | 0.016 |
| sc3 | 0.077 | 0.006 | <0.001 | 0.066 | 0.089 | 0.076 | 0.006 | <0.001 | 0.064 | 0.088 |
| sc4 | 0.202 | 0.006 | <0.001 | 0.191 | 0.214 | 0.202 | 0.006 | <0.001 | 0.189 | 0.214 |
| sc5 | 0.289 | 0.006 | <0.001 | 0.277 | 0.300 | 0.298 | 0.006 | <0.001 | 0.286 | 0.310 |
| ua2 | 0.011 | 0.006 | 0.045 | <0.001 | 0.022 | 0.008 | 0.006 | 0.144 | -0.003 | 0.020 |
| ua3 | 0.078 | 0.006 | <0.001 | 0.067 | 0.089 | 0.078 | 0.006 | <0.001 | 0.066 | 0.090 |
| ua4 | 0.207 | 0.006 | <0.001 | 0.195 | 0.218 | 0.208 | 0.006 | <0.001 | 0.196 | 0.220 |
| ua5 | 0.300 | 0.006 | <0.001 | 0.288 | 0.311 | 0.309 | 0.006 | <0.001 | 0.298 | 0.321 |
| pd2 | 0.041 | 0.005 | <0.001 | 0.030 | 0.052 | 0.038 | 0.006 | <0.001 | 0.027 | 0.049 |
| pd3 | 0.116 | 0.006 | <0.001 | 0.104 | 0.128 | 0.116 | 0.006 | <0.001 | 0.103 | 0.128 |
| pd4 | 0.320 | 0.006 | <0.001 | 0.308 | 0.332 | 0.323 | 0.006 | <0.001 | 0.311 | 0.335 |
| pd5 | 0.518 | 0.006 | <0.001 | 0.505 | 0.530 | 0.530 | 0.007 | <0.001 | 0.517 | 0.543 |
| ad2 | 0.012 | 0.006 | 0.031 | 0.001 | 0.023 | 0.010 | 0.006 | 0.094 | -0.002 | 0.021 |
| ad3 | 0.075 | 0.006 | <0.001 | 0.063 | 0.087 | 0.071 | 0.006 | <0.001 | 0.059 | 0.084 |
| ad4 | 0.230 | 0.006 | <0.001 | 0.219 | 0.242 | 0.230 | 0.006 | <0.001 | 0.218 | 0.242 |
| ad5 | 0.393 | 0.006 | <0.001 | 0.381 | 0.405 | 0.401 | 0.006 | <0.001 | 0.389 | 0.413 |
| _cons |  |  |  |  |  |  |  |  |  |  |

| **Variable** | **Linear corrected for heteroskedasticity**  **(Model 29)** | | | | | **Tobit corrected for heteroskedasticity**  **(Model 30)** | | | | |
| --- | --- | --- | --- | --- | --- | --- | --- | --- | --- | --- |
|  | **Coefficient** | **SE** | **p-value** | **95%CI** | | **Coefficient** | **SE** | **p-value** | **95%CI** | |
| mo2 | 0.001 | 0.006 | 0.887 | -0.011 | 0.013 | -0.006 | 0.006 | 0.324 | -0.018 | 0.006 |
| mo3 | 0.083 | 0.006 | <0.001 | 0.071 | 0.096 | 0.078 | 0.006 | <0.001 | 0.065 | 0.090 |
| mo4 | 0.205 | 0.007 | <0.001 | 0.192 | 0.218 | 0.202 | 0.007 | <0.001 | 0.189 | 0.215 |
| mo5 | 0.359 | 0.006 | <0.001 | 0.347 | 0.372 | 0.364 | 0.007 | <0.001 | 0.350 | 0.377 |
| sc2 | 0.008 | 0.006 | 0.159 | -0.003 | 0.019 | 0.006 | 0.006 | 0.276 | -0.005 | 0.018 |
| sc3 | 0.087 | 0.006 | <0.001 | 0.074 | 0.099 | 0.087 | 0.006 | <0.001 | 0.075 | 0.099 |
| sc4 | 0.198 | 0.007 | <0.001 | 0.185 | 0.211 | 0.200 | 0.007 | <0.001 | 0.186 | 0.213 |
| sc5 | 0.289 | 0.006 | <0.001 | 0.277 | 0.302 | 0.300 | 0.007 | <0.001 | 0.287 | 0.313 |
| ua2 | 0.003 | 0.006 | 0.605 | -0.008 | 0.014 | **-0.002** | 0.006 | 0.694 | -0.014 | 0.009 |
| ua3 | 0.074 | 0.006 | <0.001 | 0.062 | 0.085 | 0.074 | 0.006 | <0.001 | 0.062 | 0.086 |
| ua4 | 0.200 | 0.007 | <0.001 | 0.187 | 0.213 | 0.198 | 0.007 | <0.001 | 0.185 | 0.212 |
| ua5 | 0.294 | 0.006 | <0.001 | 0.282 | 0.306 | 0.301 | 0.006 | <0.001 | 0.289 | 0.314 |
| pd2 | 0.039 | 0.006 | <0.001 | 0.028 | 0.050 | 0.038 | 0.006 | <0.001 | 0.027 | 0.048 |
| pd3 | 0.121 | 0.006 | <0.001 | 0.109 | 0.133 | 0.122 | 0.006 | <0.001 | 0.109 | 0.134 |
| pd4 | 0.313 | 0.006 | <0.001 | 0.300 | 0.325 | 0.318 | 0.006 | <0.001 | 0.305 | 0.330 |
| pd5 | 0.525 | 0.007 | <0.001 | 0.512 | 0.538 | 0.539 | 0.007 | <0.001 | 0.525 | 0.553 |
| ad2 | 0.010 | 0.006 | 0.078 | -0.001 | 0.021 | 0.011 | 0.006 | 0.052 | <0.001 | 0.023 |
| ad3 | 0.078 | 0.006 | <0.001 | 0.065 | 0.091 | 0.076 | 0.007 | <0.001 | 0.063 | 0.089 |
| ad4 | 0.224 | 0.006 | <0.001 | 0.212 | 0.236 | 0.225 | 0.006 | <0.001 | 0.212 | 0.237 |
| ad5 | 0.384 | 0.006 | <0.001 | 0.372 | 0.397 | 0.392 | 0.007 | <0.001 | 0.379 | 0.405 |
| _cons |  |  |  |  |  |  |  |  |  |  |

1. **Hybrid models without flagged responses and intercept constrained (Model 31-34)**

| **Variable** | **Linear (Model 31)** | | | | | **Tobit (Model 32)** | | | | |
| --- | --- | --- | --- | --- | --- | --- | --- | --- | --- | --- |
|  | **Coefficient** | **SE** | **p-value** | **95%CI** | | **Coefficient** | **SE** | **p-value** | **95%CI** | |
| mo2 | 0.021 | 0.006 | <0.001 | 0.010 | 0.033 | 0.017 | 0.006 | 0.004 | 0.005 | 0.029 |
| mo3 | 0.087 | 0.006 | <0.001 | 0.075 | 0.098 | 0.083 | 0.006 | <0.001 | 0.071 | 0.095 |
| mo4 | 0.217 | 0.006 | <0.001 | 0.205 | 0.229 | 0.215 | 0.006 | <0.001 | 0.203 | 0.228 |
| mo5 | 0.354 | 0.006 | <0.001 | 0.342 | 0.365 | 0.359 | 0.006 | <0.001 | 0.347 | 0.372 |
| sc2 | 0.006 | 0.006 | 0.287 | -0.005 | 0.017 | 0.003 | 0.006 | 0.611 | -0.008 | 0.014 |
| sc3 | 0.077 | 0.006 | <0.001 | 0.065 | 0.088 | 0.075 | 0.006 | <0.001 | 0.063 | 0.087 |
| sc4 | 0.204 | 0.006 | <0.001 | 0.192 | 0.216 | 0.203 | 0.006 | <0.001 | 0.191 | 0.216 |
| sc5 | 0.286 | 0.006 | <0.001 | 0.274 | 0.297 | 0.295 | 0.006 | <0.001 | 0.283 | 0.307 |
| ua2 | 0.011 | 0.006 | 0.043 | <0.001 | 0.022 | 0.009 | 0.006 | 0.130 | -0.003 | 0.020 |
| ua3 | 0.078 | 0.006 | <0.001 | 0.067 | 0.090 | 0.078 | 0.006 | <0.001 | 0.067 | 0.090 |
| ua4 | 0.206 | 0.006 | <0.001 | 0.194 | 0.218 | 0.207 | 0.006 | <0.001 | 0.195 | 0.219 |
| ua5 | 0.299 | 0.006 | <0.001 | 0.288 | 0.311 | 0.308 | 0.006 | <0.001 | 0.296 | 0.320 |
| pd2 | 0.042 | 0.005 | <0.001 | 0.032 | 0.053 | 0.040 | 0.006 | <0.001 | 0.029 | 0.051 |
| pd3 | 0.118 | 0.006 | <0.001 | 0.107 | 0.130 | 0.118 | 0.006 | <0.001 | 0.106 | 0.130 |
| pd4 | 0.324 | 0.006 | <0.001 | 0.312 | 0.335 | 0.326 | 0.006 | <0.001 | 0.314 | 0.338 |
| pd5 | 0.520 | 0.006 | <0.001 | 0.507 | 0.532 | 0.531 | 0.007 | <0.001 | 0.518 | 0.544 |
| ad2 | 0.015 | 0.006 | 0.008 | 0.004 | 0.026 | 0.013 | 0.006 | 0.028 | 0.001 | 0.024 |
| ad3 | 0.078 | 0.006 | <0.001 | 0.066 | 0.090 | 0.075 | 0.006 | <0.001 | 0.063 | 0.087 |
| ad4 | 0.233 | 0.006 | <0.001 | 0.221 | 0.245 | 0.233 | 0.006 | <0.001 | 0.221 | 0.245 |
| ad5 | 0.393 | 0.006 | <0.001 | 0.382 | 0.405 | 0.401 | 0.006 | <0.001 | 0.389 | 0.413 |
| _cons |  |  |  |  |  |  |  |  |  |  |

| **Variable** | **Linear corrected for heteroskedasticity**  **(Model 33)** | | | | | **Tobit corrected for heteroskedasticity**  **(Model 34)** | | | | |
| --- | --- | --- | --- | --- | --- | --- | --- | --- | --- | --- |
|  | **Coefficient** | **SE** | **p-value** | **95%CI** | | **Coefficient** | **SE** | **p-value** | **95%CI** | |
| mo2 | 0.004 | 0.006 | 0.517 | -0.008 | 0.016 | **-0.003** | 0.006 | 0.660 | -0.015 | 0.009 |
| mo3 | 0.085 | 0.006 | <0.001 | 0.073 | 0.097 | 0.080 | 0.006 | <0.001 | 0.067 | 0.092 |
| mo4 | 0.209 | 0.006 | <0.001 | 0.196 | 0.221 | 0.206 | 0.007 | <0.001 | 0.193 | 0.219 |
| mo5 | 0.364 | 0.006 | <0.001 | 0.351 | 0.377 | 0.368 | 0.007 | <0.001 | 0.355 | 0.381 |
| sc2 | 0.007 | 0.006 | 0.243 | -0.005 | 0.018 | 0.005 | 0.006 | 0.410 | -0.007 | 0.016 |
| sc3 | 0.085 | 0.006 | <0.001 | 0.073 | 0.097 | 0.086 | 0.006 | <0.001 | 0.073 | 0.098 |
| sc4 | 0.200 | 0.007 | <0.001 | 0.186 | 0.213 | 0.201 | 0.007 | <0.001 | 0.188 | 0.215 |
| sc5 | 0.286 | 0.006 | <0.001 | 0.274 | 0.299 | 0.297 | 0.007 | <0.001 | 0.284 | 0.310 |
| ua2 | 0.003 | 0.006 | 0.631 | -0.009 | 0.014 | **-0.003** | 0.006 | 0.655 | -0.014 | 0.009 |
| ua3 | 0.073 | 0.006 | <0.001 | 0.061 | 0.085 | 0.073 | 0.006 | <0.001 | 0.061 | 0.085 |
| ua4 | 0.199 | 0.007 | <0.001 | 0.186 | 0.212 | 0.198 | 0.007 | <0.001 | 0.185 | 0.211 |
| ua5 | 0.294 | 0.006 | <0.001 | 0.283 | 0.306 | 0.302 | 0.006 | <0.001 | 0.290 | 0.314 |
| pd2 | 0.040 | 0.006 | <0.001 | 0.029 | 0.050 | 0.039 | 0.006 | <0.001 | 0.028 | 0.049 |
| pd3 | 0.123 | 0.006 | <0.001 | 0.111 | 0.135 | 0.123 | 0.006 | <0.001 | 0.111 | 0.136 |
| pd4 | 0.315 | 0.006 | <0.001 | 0.302 | 0.327 | 0.320 | 0.007 | <0.001 | 0.307 | 0.332 |
| pd5 | 0.525 | 0.007 | <0.001 | 0.512 | 0.538 | 0.538 | 0.007 | <0.001 | 0.524 | 0.552 |
| ad2 | 0.012 | 0.006 | 0.036 | 0.001 | 0.023 | 0.013 | 0.006 | 0.023 | 0.002 | 0.025 |
| ad3 | 0.081 | 0.006 | <0.001 | 0.068 | 0.093 | 0.079 | 0.007 | <0.001 | 0.066 | 0.092 |
| ad4 | 0.226 | 0.006 | <0.001 | 0.214 | 0.238 | 0.227 | 0.006 | <0.001 | 0.215 | 0.239 |
| ad5 | 0.385 | 0.006 | <0.001 | 0.373 | 0.398 | 0.393 | 0.007 | <0.001 | 0.380 | 0.406 |
| _cons |  |  |  |  |  |  |  |  |  |  |

**Supplementary Table 2:** Model performance and comparison of order of importance of the candidate models

| **Model type (Model number)** | **AIC** | **BIC** | **MAE** | **Order of importance dimensions** |
| --- | --- | --- | --- | --- |
| TTO model with all responses and intercept (Model 4) | 860 | 1171 | 0.0612 | PD>AD>MO>SC>UA |
| TTO model with all responses without intercept (Model 12) | 878 | 1181 | 0.0625 | PD>AD>MO>SC>UA |
| TTO model without flagged responses with intercept (Model 8) | 516 | 824 | 0.0619 | PD>AD>MO>SC>UA |
| TTO model without flagged responses and without intercept (Model 16) | 531 | 833 | 0.0632 | PD>AD>MO>SC>UA |
|  |  |  |  |  |
| Hybrid model with all responses included and intercept (Model 22) | 20089 | 20441 | 0.0737 | PD>AD>MO>UA>SC |
| Hybrid model with all responses included without intercept (Model 30) | 26027 | 26362 | 0.0682 | PD>AD>MO>UA>SC |
| Hybrid model without flagged responses with intercept (Model 26) | 19623 | 19974 | 0.0647 | PD>AD>MO>UA>SC |
| Hybrid model without flagged responses without intercept (Model 34) | 25555 | 25890 | 0.0671 | PD>AD>MO>UA>SC |
|  |  |  |  |  |
| DCE Conditional Logit Model (Model 17) | - | - | - | PD>MO>AD>UA>SC |
| DCE Mixed Logit Model (Model 18) | - | - | - | PD>AD>MO>UA>SC |

AIC: Akaike Information Criterion, BIC: Bayesian Information Criterion, DCE: Discrete Choice Experiment, MAE: Mean Absolute Error, MO: mobility, SC: self-care, UA: usual activities, PD: pain/discomfort, AD: Anxiety/depression, TTO: time-trade off, SE: Standard error.

**Supplementary Table 3:** Model comparison between Hausa and English versions of the EQ-PVT

| **Coefficient for Hausa version (Model 35) *** | **Coefficient** | **SE** | **P- Value** | **95% Confidence Interval** | |
| --- | --- | --- | --- | --- | --- |
| mo2 | 0.020 | 0.006 | <0.01 | 0.008 | 0.031 |
| mo3 | 0.051 | 0.010 | <0.01 | 0.032 | 0.070 |
| mo4 | 0.236 | 0.011 | <0.01 | 0.215 | 0.257 |
| mo5 | 0.303 | 0.009 | <0.01 | 0.285 | 0.321 |
| sc2 | 0.032 | 0.005 | <0.01 | 0.022 | 0.043 |
| sc3 | 0.070 | 0.008 | <0.01 | 0.056 | 0.085 |
| sc4 | 0.249 | 0.010 | <0.01 | 0.229 | 0.269 |
| sc5 | 0.262 | 0.008 | <0.01 | 0.246 | 0.279 |
| ua2 | 0.035 | 0.005 | <0.01 | 0.024 | 0.045 |
| ua3 | 0.079 | 0.008 | <0.01 | 0.064 | 0.095 |
| ua4 | 0.195 | 0.009 | <0.01 | 0.178 | 0.212 |
| ua5 | 0.249 | 0.009 | <0.01 | 0.231 | 0.266 |
| pd2 | 0.042 | 0.005 | <0.01 | 0.033 | 0.051 |
| pd3 | 0.060 | 0.009 | <0.01 | 0.043 | 0.077 |
| pd4 | 0.281 | 0.009 | <0.01 | 0.263 | 0.299 |
| pd5 | 0.472 | 0.010 | <0.01 | 0.452 | 0.492 |
| ad2 | 0.040 | 0.005 | <0.01 | 0.031 | 0.049 |
| ad3 | 0.075 | 0.009 | <0.01 | 0.057 | 0.093 |
| ad4 | 0.216 | 0.008 | <0.01 | 0.200 | 0.233 |
| ad5 | 0.355 | 0.008 | <0.01 | 0.339 | 0.370 |
| Δcons | 0.024 | 0.013 | 0.058 | -0.001 | 0.050 |
| **Coefficients for Hausa – Coefficients for English** | | | | | |
| Δmo2 | -0.013 | 0.015 | 0.385 | -0.041 | 0.016 |
| Δmo3 | -0.005 | 0.025 | 0.844 | -0.055 | 0.045 |
| Δmo4 | -0.066 | 0.028 | 0.020 | -0.121 | -0.011 |
| Δmo5 | -0.048 | 0.023 | 0.038 | -0.093 | -0.003 |
| Δsc2 | -0.004 | 0.014 | 0.783 | -0.030 | 0.023 |
| Δsc3 | -0.009 | 0.020 | 0.645 | -0.049 | 0.030 |
| Δsc4 | -0.030 | 0.026 | 0.246 | -0.081 | 0.021 |
| Δsc5 | -0.031 | 0.022 | 0.151 | -0.074 | 0.011 |
| Δua2 | -0.009 | 0.014 | 0.506 | -0.036 | 0.018 |
| Δua3 | 0.003 | 0.021 | 0.872 | -0.038 | 0.045 |
| Δua4 | 0.031 | 0.023 | 0.186 | -0.015 | 0.076 |
| Δua5 | 0.001 | 0.022 | 0.965 | -0.043 | 0.045 |
| Δpd2 | -0.030 | 0.012 | 0.013 | -0.054 | -0.006 |
| Δpd3 | -0.019 | 0.023 | 0.403 | -0.065 | 0.026 |
| Δpd4 | -0.082 | 0.023 | <0.01 | -0.127 | -0.036 |
| Δpd5 | -0.048 | 0.027 | 0.072 | -0.100 | 0.004 |
| Δad2 | -0.003 | 0.012 | 0.793 | -0.026 | 0.020 |
| Δad3 | -0.002 | 0.023 | 0.943 | -0.047 | 0.044 |
| Δad4 | -0.001 | 0.022 | 0.948 | -0.045 | 0.042 |
| Δad5 | 0.036 | 0.020 | 0.078 | -0.004 | 0.075 |
| Δcons | 0.017 | 0.005 | <0.01 | 0.007 | 0.027 |

* Tobit corrected for heteroskedasticity with intercept, mo: mobility, sc: self-care, ua: usual activities, pd: pain/discomfort, ad: Anxiety/depression, SE: Standard error.

**Supplementary Table 4:** Mean cTTO Values of the 86 health states

| Health State | Mean | Standard Error | Health State | Mean | Standard Error |
| --- | --- | --- | --- | --- | --- |
| 11112 | 0.955 | 0.003 | **31524** | 0.447 | 0.028 |
| 11121 | 0.952 | 0.003 | **31525** | 0.322 | 0.031 |
| 11122 | 0.898 | 0.006 | **32314** | 0.605 | 0.015 |
| 11211 | 0.951 | 0.003 | **32443** | 0.356 | 0.028 |
| 11212 | 0.879 | 0.007 | **33253** | 0.320 | 0.030 |
| 11221 | 0.868 | 0.007 | **34155** | -0.227 | 0.040 |
| 11235 | 0.526 | 0.020 | **34232** | 0.607 | 0.015 |
| 11414 | 0.604 | 0.019 | **34244** | 0.090 | 0.038 |
| 11421 | 0.750 | 0.010 | **34515** | 0.011 | 0.039 |
| 11425 | 0.472 | 0.021 | **35143** | 0.366 | 0.031 |
| 12111 | 0.948 | 0.005 | **35245** | -0.163 | 0.041 |
| 12112 | 0.869 | 0.008 | **35311** | 0.599 | 0.017 |
| 12121 | 0.874 | 0.009 | **35332** | 0.463 | 0.029 |
| 12244 | 0.473 | 0.020 | **42115** | 0.440 | 0.025 |
| 12334 | 0.579 | 0.015 | **42321** | 0.655 | 0.017 |
| 12344 | 0.384 | 0.026 | **43315** | 0.304 | 0.030 |
| 12513 | 0.630 | 0.016 | **43514** | 0.196 | 0.035 |
| 12514 | 0.529 | 0.018 | **43542** | 0.115 | 0.039 |
| 12543 | 0.428 | 0.025 | **43555** | -0.533 | 0.034 |
| 13122 | 0.789 | 0.009 | **44125** | 0.101 | 0.038 |
| 13224 | 0.645 | 0.013 | **44345** | -0.412 | 0.034 |
| 13313 | 0.677 | 0.013 | **44553** | -0.449 | 0.039 |
| 14113 | 0.721 | 0.011 | **45133** | 0.370 | 0.030 |
| 14554 | -0.347 | 0.041 | **45144** | -0.187 | 0.043 |
| 15151 | 0.362 | 0.033 | **45233** | 0.330 | 0.031 |
| 21111 | 0.948 | 0.004 | **45413** | 0.175 | 0.037 |
| 21112 | 0.883 | 0.008 | **51152** | 0.260 | 0.032 |
| 21315 | 0.572 | 0.017 | **51451** | -0.056 | 0.042 |
| 21334 | 0.589 | 0.014 | **52215** | 0.305 | 0.032 |
| 21345 | 0.281 | 0.028 | **52335** | 0.166 | 0.036 |
| 21444 | 0.095 | 0.042 | **52431** | 0.418 | 0.028 |
| 22434 | 0.480 | 0.021 | **52455** | -0.532 | 0.033 |
| 23152 | 0.448 | 0.022 | **53221** | 0.613 | 0.016 |
| 23242 | 0.594 | 0.018 | **53243** | 0.262 | 0.036 |
| 23514 | 0.475 | 0.021 | **53244** | 0.035 | 0.043 |
| 24342 | 0.432 | 0.025 | **53412** | 0.439 | 0.028 |
| 24443 | 0.127 | 0.039 | **54153** | -0.062 | 0.045 |
| 24445 | -0.333 | 0.036 | **54231** | 0.420 | 0.025 |
| 24553 | -0.161 | 0.040 | **54342** | 0.048 | 0.043 |
| 25122 | 0.667 | 0.017 | **55225** | -0.176 | 0.038 |
| 25222 | 0.702 | 0.021 | **55233** | 0.206 | 0.035 |
| 25331 | 0.532 | 0.027 | **55424** | -0.344 | 0.042 |
| 31514 | 0.478 | 0.023 | **55555** | -0.865 | 0.005 |

**Supplementary Table 5:** Comparison demographic characteristics between the English and Hausa samples

| **Variable** | **English (n=1,022)** | **Hausa (n=175)** | **P-Value** |
| --- | --- | --- | --- |
|  | **n(%)** | **n(%)** |  |
| **Gender** |  |  | 0.530 |
| Male | 511(50.0) | 83(47.3) |  |
| Female | 511(50.0) | 92(52.6) |  |
| **Age** |  |  |  |
| Mean | 36.1 | 41.1 |  |
| Median | 33 | 35 |  |
| SD | 15.6 | 18.4 |  |
| IQR | 23 | 31 |  |
| **Age group (years)** |  |  | 0.001 |
| 18-24 | 321(31.4) | 49(28.0) |  |
| 25-34 | 230(22.5) | 26(14.9) |  |
| 35-44 | 190(18.6) | 30(17.1) |  |
| 45-54 | 124(12.1) | 24(13.7) |  |
| 55-56 | 79(7.7) | 16(9.1) |  |
| 65-74 | 52(5.1) | 17(9.7) |  |
| 75+ | 26(2.5) | 13(7.4) |  |
| **Education** |  |  | <0.001 |
| No School | 157(15.4) | 56(32.0) |  |
| Primary | 227(22.2) | 25(14.3) |  |
| Secondary | 362(35.4) | 26(14.9) |  |
| Tertiary | 145(14.2) | 5(2.9) |  |
| Religious/Others | 131(12.8) | 63(36.0) |  |
| **Ethnicity** |  |  |  |
| Hausa | 190(18.6) | 151(86.3) | <0.001 |
| Igbo | 178(17.4) | 1(0.57) |  |
| Yoruba | 178(17.4) | 0(0.0) |  |
| Others | 476(46.6) | 23(13.1) |  |
| **Religion** |  |  | <0.001* |
| Christianity | 573(56.1) | 3(1.7) |  |
| Islam | 444(43.4) | 172(98.3) |  |
| Traditional | 5(0.49) | 0(0.0) |  |
| **Occupation** |  |  | <0.001 |
| Housewife | 60(5.9) | 44(25.1) |  |
| Student | 136(13.3) | 11(6.3) |  |
| Retired | 22(2.2) | 3(1.7) |  |
| Not working | 60(5.9) | 9(5.1) |  |
| Employed | 105(10.3) | 12(6.9) |  |
| Self employed | 639(62.5) | 96(54.9) |  |
| **Marital status** |  |  | <0.001* |
| Single | 469(45.9) | 44(25.1) |  |
| Married | 509(49.8) | 123(70.3) |  |
| Divorced | 12(1.2) | 2(1.1) |  |
| Widowed | 29(2.8) | 6(3.4) |  |
| Others | 3(0.3) | 0(0.0) |  |

*= Fisher’s Exact Chi-Square test


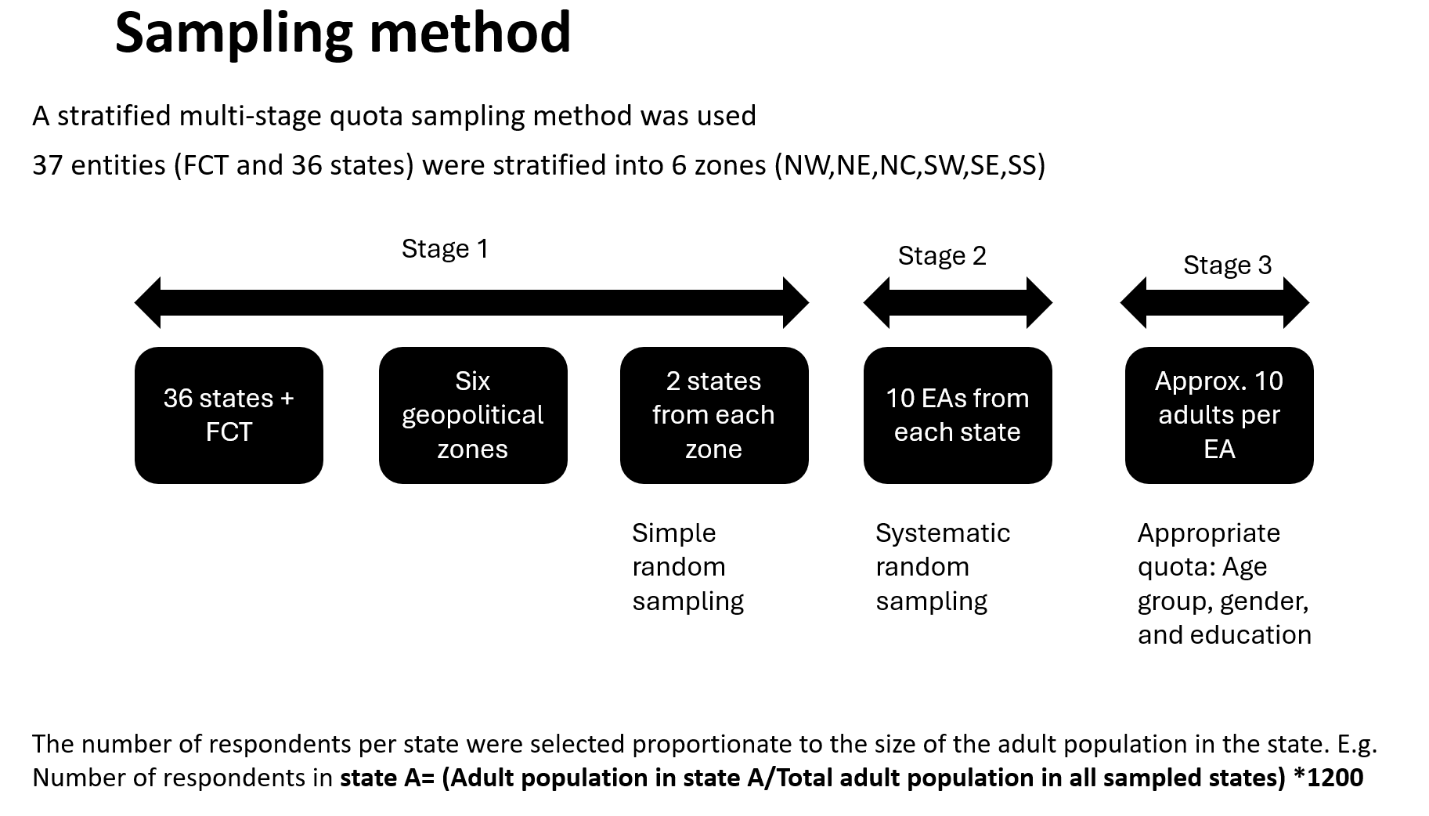


**Supplement figure 1:** Sampling strategy

**Supplementary Figure 2:** Scatter plots for the agreement between cTTO and DCE models

**Supplementary figure 3:** Bland-Altman plot for the agreement between the cTTO and the DCE models
